# Brazilian Consensus Statement on Lipedema using the Delphi methodology

**DOI:** 10.1590/1677-5449.202301832

**Published:** 2025-02-10

**Authors:** Alexandre Campos Moraes Amato, Ana Paula Rolim Maia Peclat, Rodrigo Kikuchi, Antonio Carlos de Souza, Mariana Thalyta Bertolin Silva, Roney Hans Prager de Oliveira, Daniel Augusto Benitti, Julio Cesar Peclat de Oliveira

**Affiliations:** 1 Sociedade Brasileira de Angiologia e de Cirurgia Vascular – SBACV, São Paulo, SP, Brasil.; 2 Amato – Instituto de Medicina Avançada, São Paulo, SP, Brasil.; 3 Faculdade Souza Marques – FTESM, Rio de Janeiro, RJ, Brasil.; 4 Clínica Peclat, São Gonçalo, RJ, Brasil.; 5 Santa Casa de São Paulo, São Paulo, SP, Brasil.; 6 Instituto de Excelência Vascular, Londrina, PR, Brasil.

**Keywords:** lipedema, obesity, lymphoedema, consensus

## Abstract

Lipedema, historically underrecognized, has recently gained attention due to advancements in research and growing public awareness. The Brazilian Consensus Statement on Lipedema, developed by the Brazilian Society of Angiology and Vascular Surgery, aims to establish clear recommendations for the diagnosis, treatment, and management of lipedema. Using the Delphi methodology, experts elaborated 90 statements about lipedema, which were then evaluated by a panel of 113 professionals. The statements were analyzed using SurveyMonkey, with a 75% agreement threshold required for their inclusion in the consensus statement. Most statements achieved significant consensus, with only 9 topics requiring further investigation. This consensus statement highlights the complexity of lipedema, the effectiveness of conservative treatment over surgery, the need for multidisciplinary approaches, and the importance of awareness to reduce underdiagnosis and stigma. It also underscores the ongoing need for research to develop more effective management strategies.

## INTRODUCTION

Lipedema is a clinical condition that has been recognized for decades.^1^ However, it remains underdiagnosed and misunderstood, despite Brazil being a pioneer in its surgical treatment through the work of Professor Ivo Pitanguy.^2^ Until now, this condition had remained relatively obscure in both the international and national medical literature,^3^ as well as in public awareness. Recently, however, interest in lipedema has increased due to advances in medical research, new surgical techniques, and increased awareness among both patients and health care professionals. This renewed interest promoted the need for clear recommendations on the identification, management, and treatment of lipedema. The objective of this article is to report the results of the Brazilian Consensus Statement on Lipedema, conducted by the Brazilian Society of Angiology and Vascular Surgery (SBACV). This multidisciplinary effort aimed to clarify uncertainties, establish clinical parameters, and guide the approach to lipedema in Brazil, promoting a deeper understanding and more effective treatment of this condition.

Lipedema is a complex condition that affects several dimensions of women’s health, challenging current clinical and therapeutic beliefs.^4^ Its complexity has contributed to a fragmented clinical landscape, in which the absence of an established consensus has led to the emergence of individual protocols and a frantic pursuit of treatments. In this setting, health care professionals often offer solutions based on their personal experiences and individual perspectives rather than on evidence-based approaches supported by scientific knowledge and expert consensus.

This phenomenon is particularly evident in the surgical approach to lipedema. Surgical procedures are often considered the solution, but they still lack rigorous validation and consensus among the medical community.^5^ The lack of a comprehensive and integrated understanding of the pathophysiology of lipedema adds another layer of complexity, limiting the ability of health care professionals to deliver effective, evidence-based treatments to patients.

However, despite these uncertainties and challenges, patients with lipedema exist, face difficulties in their daily lives, and demand urgent solutions.^4^ The need for answers to their current problems is a tangible and pressing reality. In this context, the development of a consensus statement is not only desirable but extremely necessary. A consensus statement can provide common ground, a foundation upon which protocols for diagnosis, treatment, and management can be built. In addition, it can guide health care professionals in their clinical practice and offer patients a clearer and safer path for the management of their condition. Therefore, we found that a consensus statement on lipedema was urgent and crucial to advance the care and understanding of this challenging condition.

The Delphi technique, developed in the 1950s at the RAND Corporation by Norman C. Dalkey,^6^ is a well-established method for achieving expert consensus in specific fields. The technique is based on the principle that decisions from a structured group of specialists are more accurate than those from unstructured groups or individuals. The Delphi method is notable for its straightforward process of collecting and examining opinions while maintaining the anonymity of participants (their identities are not revealed even after completion of the final report), and for allowing geographical spread of the participants in a cost-effective and efficient manner.^7^

To avoid common problems associated with group discussions, such as peer influence or pressure to conform to the majority, interactions between participants are limited.^6^ The experts answer questionnaires in several rounds and, after each round, a facilitator provides an anonymized summary of their responses.^6-8^ This allows participants to revise their earlier statements in light of the other responses. Finally, statistical analyses are conducted to provide an objective and impartial evaluation of the data, ensuring that all opinions are adequately represented in the final iteration.^6-8^

The aim of this study was to develop, in Phase 1, a set of statements on lipedema by an expert panel. These statements were then validated and revised in Phases 2 and 3 until broad consensus was reached. This thorough process ensured that the final statements reflected an evidence-based collective agreement among participants, addressing the validity, clarity, and relevance of the statements.

## METHODS

All participants signed an informed consent form. The study was conducted in accordance with the standards established by Resolution No. 196/96 of the Brazilian National Health Council regarding research involving humans, as well as the Declaration of Helsinki.

### Phase 1

**Figure 1:**
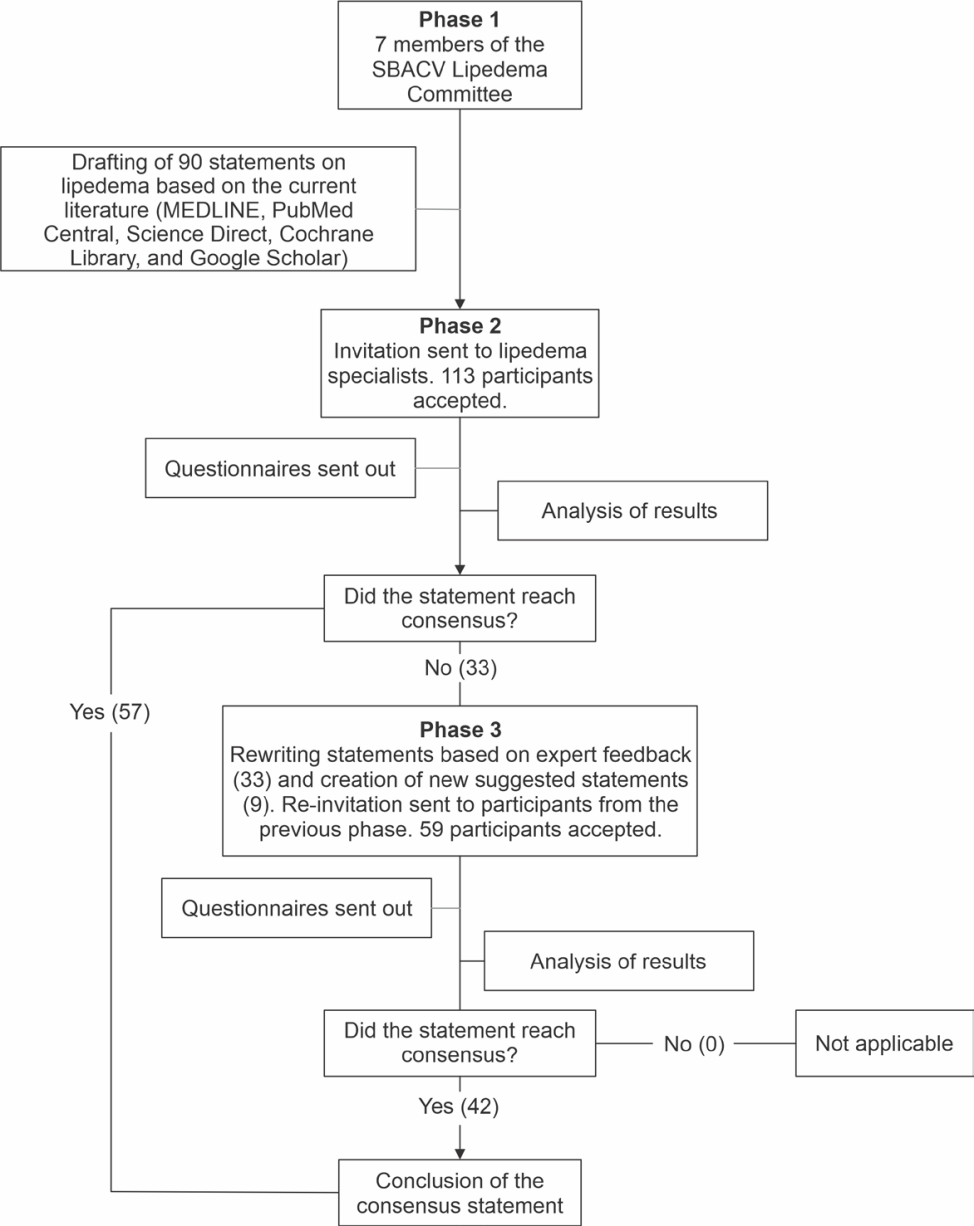
Flowchart of participants and the Delphi technique.

In the first phase of the consensus statement, 7 participants were selected, each representing their respective specialties. All were members of the 2022/2023 Lipedema Committee of the SBACV and had proven expertise in the field, with a minimum of 8 years of experience and relevant publications. They were chosen from among leading specialists and active members within the society, reflecting the range of knowledge and approaches necessary for a comprehensive understanding of lipedema. Each specialist, distributed across various regions of Brazil, contributed their unique expertise to the study (Figure 1).

The main objective of Phase 1 was to establish the foundation for the consensus statement. To achieve this, each participant individually searched the MEDLINE, PubMed Central, Science Direct, Cochrane Library, and Google Scholar databases for publications on lipedema. This literature review focused on identifying the key aspects, challenges, and gaps in knowledge related to lipedema.

Based on this review, participants developed a series of statements covering multiple topics related to lipedema, such as diagnosis, treatment, and management. Each statement was evaluated in subsequent phases of the study for clarity, relevance, and scientific support.

To foster communication and collaboration among participants, a group was created on an instant messaging platform, which served as a forum for open discussions, exchange of ideas, and refinement of statements. It is worth noting that no formal measurement of participants’ opinions was conducted during this phase; the focus remained on group discussion and drafting of the statements, laying the foundation for more structured evaluations in subsequent phases.

Thus, Phase 1 laid the foundation for the consensus statement, ensuring that the statements to be evaluated in Phases 2 and 3 were comprehensive, relevant, and reflected a multidisciplinary understanding of lipedema. The topics addressed included etiology, epidemiology, multidisciplinary approach, clinical characteristics, pathogenesis/pathophysiology, diagnosis, prognosis, social and psychological aspects, treatment, prevention, research and development, and classification.

### Phase 2

In the second phase of the consensus statement, participation was expanded to include 113 specialists from across Brazil, representing a wide range of medical specialties and related health care fields. These specialists were invited through an official SBACV email and through lipedema study groups. Unlike the first phase, which involved a smaller, highly specialized group, this phase did not require specific expertise in lipedema. However, participants had to be actively involved in the care or study of lipedema.

Using the online platform SurveyMonkey (SurveyMonkey®, San Mateo, CA, USA), a thorough evaluation of the 90 statements developed in Phase 1 was conducted. The specialists, including participants of lipedema discussion groups and SBACV members, were invited via an official SBACV email to participate. Each participant individually reviewed the statements and rated them according to four criteria: agreement with the statement, whether the statement should be included in the final report, whether the statement was clear, and whether there was sufficient evidence to support the statement.

Although participants were required to provide identifying information, all feedback on the statements was anonymized to ensure objectivity and impartiality. There was no direct interaction between participants, ensuring that opinions were independently formed without the influence of group discussions. Additionally, the specialists were given the opportunity to provide qualitative feedback on each statement and suggest new ones to be considered in the next phase of the study.

This approach allowed for the expression of different perspectives on the statements related to lipedema, contributing to the development of a more robust and representative consensus statement that reflects the wide-ranging views and experiences of professionals involved in the diagnosis and treatment of this condition.

### Phase 3

In the third phase of the consensus statement, all specialists who participated in the previous phases were reinvited, emphasizing the importance of their continued participation. Of those invited, 59 specialists actively participated in the final phase, including 51 vascular surgeons, 3 angiologists, 1 plastic surgeon, 2 physiotherapists, 1 gynecologist, and 1 nutritionist.

The statistics from Phase 2 were compiled anonymously, and the statements were re-evaluated and rewritten based on four established criteria:

1. **“Agreement with the statement”**: Statements above the 75% agreement threshold were accepted as part of the consensus.
2. **“The statement should be included in the final report”**: Statements above the 75% agreement threshold were accepted for inclusion in the consensus statement.
3. **“The statement is clear”**: Statements below the 75% agreement threshold were revised and rewritten for further validation, incorporating suggestions from participants’ feedback.
4. **“There is sufficient evidence to support the statement”**: Statements above the 75% agreement threshold were considered sufficiently evidence-based for publication.

Any statement that did not meet these four criteria was revalidated in the third phase of the study. Therefore, a statement had to meet all 4 criteria to be included in the consensus statement without being re-evaluated in Phase 3.

In Phase 3, the number of statements requiring further validation was 42, reflecting significant improvements in the content and clarity of the statements on lipedema.

### Statistical analysis

**Phase 1:** The first phase was primarily descriptive and did not include any statistical analysis. Instead, it focused on drafting and discussing the statements based on the literature review and the expertise of the participants.

**Phases 2 and 3:** During Phases 2 and 3, specific statistical analyses were conducted to evaluate the statements regarding lipedema. Participants rated each statement using a 5-point Likert scale, where 1 represented “strongly disagree” and 5 indicated “strongly agree.” The scores from all participants for each statement were summed to generate a total score.

From these total scores, a weighted average was calculated considering the number of responses for each statement. This weighted average was computed for the four criteria previously described: “Agreement with the statement,” “The statement should be included in the final report,” “The statement is clear,” and “There is sufficient evidence to support the statement.”

A threshold of 75% agreement was established based on the weighted average to determine whether a statement would be part of the consensus statement. Statements that achieved this threshold for any of the criteria were accepted for inclusion or publication. In contrast, those that did not meet the 75% threshold were revised and re-evaluated in the next phase.

## RESULTS

### Phase 1

In the first phase of the consensus statement, a total of 90 statements were developed. These statements were carefully drafted based on the collective expertise of the specialists involved and the latest literature on the subject. The resulting set of statements covered a wide range of topics related to lipedema, including diagnosis, treatment, clinical management, and pathophysiology.

### Phase 2

**Figure 2:**
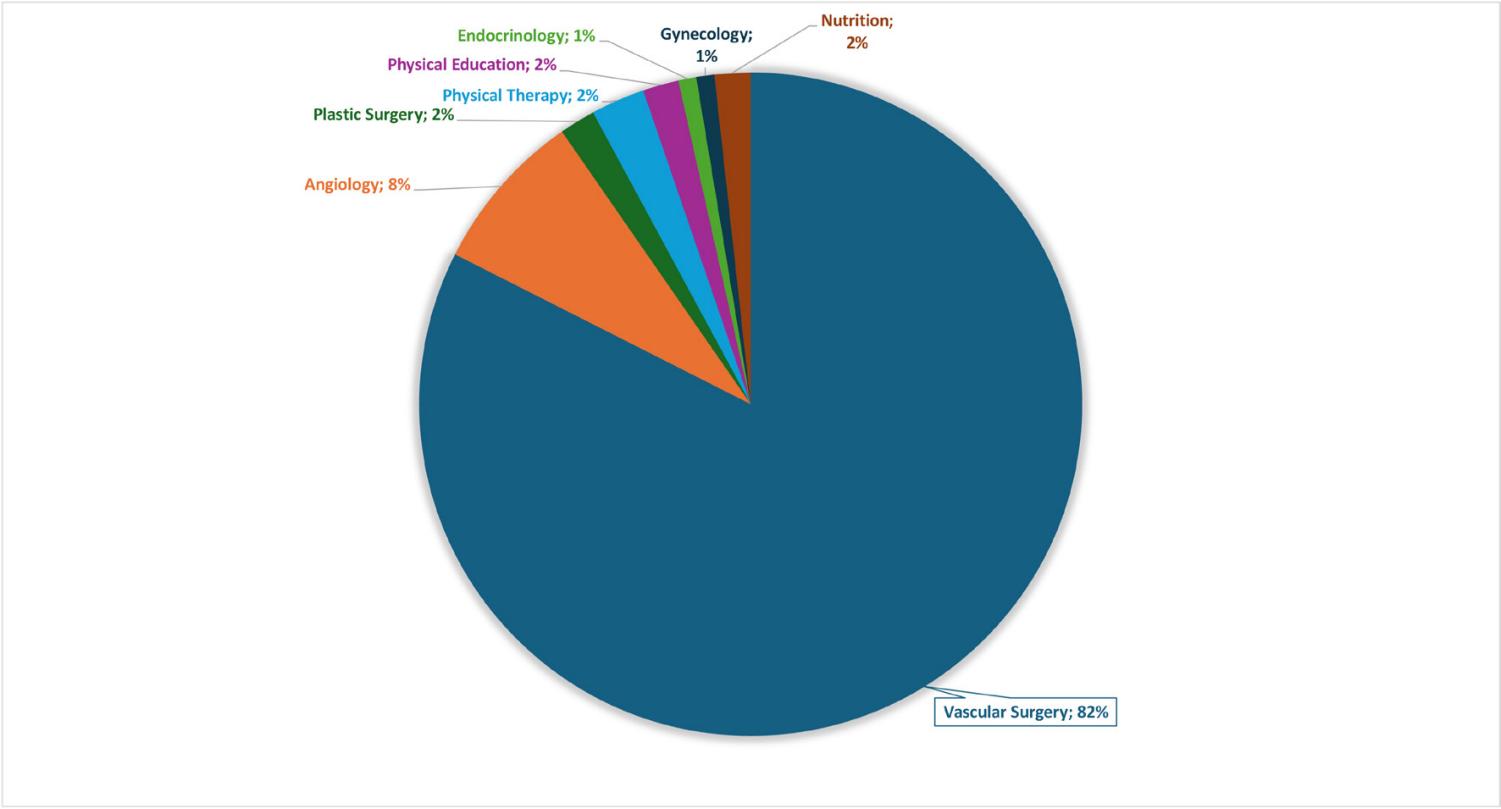
Distribution of medical specialties and health care professionals.

The second phase of the consensus statement involved 113 specialists from several health fields, who participated voluntarily and were geographically distributed across Brazil. On average, participants had approximately 21.6 years of medical practice, with 8 years of experience in treating patients with lipedema. Most participants specialized in Vascular Surgery (83.20%), followed by Angiology (8%), Plastic Surgery (1.8%), Physical therapy (2.7%), Physical Education (1.8%), Nutrition (1.8%), Endocrinology (0.9%), and Gynecology (0.9%) (Figure 2).

**Figure 3:**
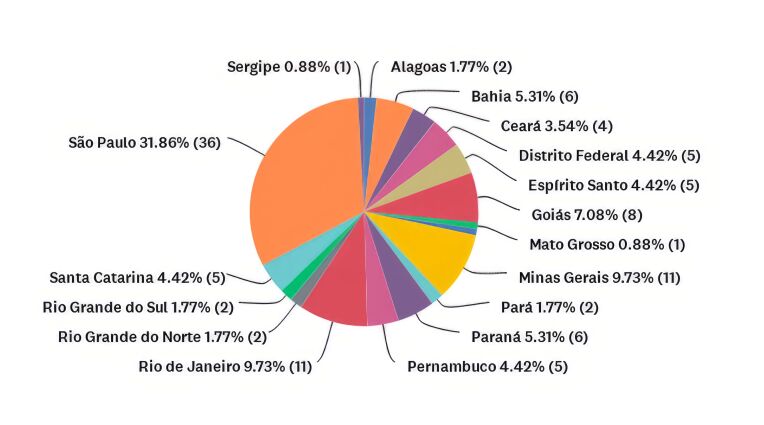
Demographic distribution of participants.

As for their geographical distribution, most participants were from São Paulo (31,86%), followed by Minas Gerais (9.73%), Rio de Janeiro (9.73%), and Bahia (5.31%). The complete geographical distribution of participants is shown in Figure 3.

Regarding their involvement in the management of lipedema, most participants (91.15%) engaged in clinical treatment. Other therapeutic approaches, such as diet (45.13%), physical activity (47.79%), and physical therapy (32.74%), were also common. A smaller percentage of participants reported working in surgery (15.93%) and diagnostic imaging (35.40%).

At the end of this phase, 33 statements failed to reach consensus or had clarity issues. These statements were selected for review and re-evaluation in Phase 3 of the study. An additional 9 statements were created based on participants’ suggestions. This refinement process was essential to ensure that the final statements were clear, accurate, and reflected a consensus among the medical community regarding the management of lipedema.

The statements that reached consensus in Phase 2 of the study are detailed in Table 1.^9-79^ Those that did not reach consensus were revised, rewritten, and subsequently re-evaluated in Phase 3.

**Table 1:** Phase 2 results.

**Statement**	**Topic**	**Agreement**	**Inclusion**	**Clarity**	**Evidence**	**Responded/skipped**	**Level of evidence**
Lipedema is a chronic, systemic disease.^9,10^	Etiology	4.31	4.19	4.2	3.96	110/3	B
The prevalence of lipedema in Brazil is 12.3% of the adult female population.^4^	Epidemiology	3.78	3.81	3.98	3.49	109/4	B
Lipedema is a complex disease that requires multidisciplinary treatment.^11,12^	Multidisciplinary approach	4.66	4.62	4.6	4.45	106/7	B
Lipedema usually presents as a symmetrical, bilateral increase in subcutaneous adipose tissue in the limbs.^13,14^	Clinical characteristics	4.2	4.17	4.13	4.04	106/7	B
Lipedema is characterized by the disproportionate distribution of subcutaneous adipose tissue in the limbs in relation to the trunk.^12,15^	Pathogenesis/pathophysiology	4.29	4.15	4.18	4.12	106/7	B
Lipedema may cause excessive, symmetrical, and bilateral deposition of adipose tissue in the upper limbs.^10,12,13^	Pathogenesis/pathophysiology	3.98	3.92	4.02	3.85	105/8	B
Lipedema typically spares the hands and feet.^14,16^	Clinical characteristics	4.32	4.26	4.34	4.19	104/9	B
Painful sensitivity to pressure and/or stretching is often observed through palpation and is commonly reported by patients as pain.^17,18^	Clinical characteristics	4.39	4.34	4.37	4.2	102/11	B
Patients frequently report swelling or a sensation of heaviness in the affected areas.^4,12,19,20^	Clinical characteristics	4.4	4.35	4.31	4.21	102/11	B
Pitting edema (Godet sign) is not usually present in tissue affected by lipedema.^12,13,21^	Diagnosis	4.12	4.08	4.14	4.05	100/13	B
Elevation of the affected limb does not tend to result in volume reduction.^22,23^	Diagnosis	4.15	4.13	4.19	4.12	99/14	B
Patients with lipedema often bruise easily.^4,12,23,24^	Clinical characteristics	4.07	4.09	4.14	3.97	99/14	B
The Kaposi-Stemmer sign is usually negative in patients with lipedema.^25,26^	Diagnosis	4.27	4.24	4.25	4.15	97/16	B
Lipedema is a disease involving subcutaneous adipose tissue.^15,27,28^	Clinical characteristics	4.29	4.21	4.22	4.13	97/16	B
Several findings suggest that inflammation may contribute to the pathogenesis of lipedema.^20,21,29^	Pathogenesis/pathophysiology	4.25	4.2	4.22	4.08	96/17	B
Several findings suggest that hormonal factors may contribute to the pathogenesis of lipedema.^9,12,30,31^	Pathogenesis/pathophysiology	4.37	4.37	4.32	4.13	95/18	B
Several findings suggest that extracellular fluid may be increased in tissue affected by lipedema compared with healthy controls.^32-34^	Pathogenesis/pathophysiology	4.1	3.98	3.98	3.89	93/20	C
Lipedema primarily affects individuals assigned female at birth. Although possible, occurrence in men is rare.^25,35,36^	Clinical characteristics	4.23	4.15	4.2	4.09	92/21	B
Hormonal changes, such as those during puberty, pregnancy, and menopause, can trigger or exacerbate lipedema symptoms.^10,12,25^	Clinical characteristics	4.46	4.41	4.46	4.34	93/20	B
Several findings suggest that lipedema is hereditary.^25,37-39^	Etiology	4.26	4.26	4.28	4.13	93/20	B
Obesity is a common comorbidity in patients with lipedema.^4,12,23^	Epidemiology	3.99	3.9	4.04	3.9	93/20	B
Lipedema and obesity do not have a causal relationship.^12,40^	Pathogenesis/pathophysiology	3.86	3.79	3.86	3.66	93/20	B
Body mass index is of limited value in differentiating lipedema from obesity.	Diagnosis	4.13	4.1	4.16	3.98	93/20	B
When lipedema coexists with obesity, its symptoms are likely to persist even after bariatric surgery.^25,40,41^	Prognosis	4.22	4.15	4.17	4.01	93/20	C
Lipedema can negatively impact mental health and overall quality of life.^4,42^	Social and psychological aspects	4.66	4.59	4.62	4.47	87/26	B
Delayed diagnosis or late treatment negatively affects symptom burden, mental well-being, and overall quality of life.^43-45^	Prognosis	4.45	4.38	4.38	4.26	87/26	C
The clinical diagnosis of lipedema relies on the patient’s medical history, physical examination, and exclusion of differential diagnoses.^23,27,46^	Diagnosis	4.44	4.41	4.44	4.32	87/26	B
All therapeutic interventions aim to relieve symptoms and prevent or slow disease progression.^11,47^	Treatment	4.14	4.15	4.2	4.04	81/32	B
Multidisciplinary care involving physicians, physical therapists, nutritionists, and mental health professionals is essential for comprehensive management. The medical specialties that should be involved in treating lipedema include Vascular Surgery, Endocrinology, Orthopedics, Plastic Surgery, Psychiatry, and Gynecology, among others.^48-50^	Multidisciplinary approach	4.48	4.46	4.46	4.39	79/34	B
Pain and sensitivity in areas affected by lipedema have been reported to be reduced by compression bandages, complex decongestive therapy, dietary changes, low-impact exercises, and liposuction, with varying effect sizes and durations.^51-55^	Treatment	4.38	4.3	4.34	4.14	79/34	C
Conservative management of lipedema should include lifestyle and dietary changes, compression therapy, and low-impact exercise to alleviate symptoms and improve quality of life.^23,38,54,55^	Treatment	4.52	4.47	4.48	4.33	79/34	C
Complex decongestive therapy, incorporating at least some of its elements, can be an important and effective treatment, even for early-stage lipedema.^56-58^	Treatment	4.26	4.18	4.25	4.08	78/35	C
Nutrition counseling can help patients manage their weight, optimize their overall health, alleviate lipedema symptoms, and improve the efficacy of other therapeutic interventions.^12,40^	Treatment	4.55	4.5	4.53	4.42	78/35	C
Considering that severe obesity exacerbates lipedema symptoms, disease management should include weight normalization, focusing on the waist-to-height ratio.^59,60^	Treatment	3.98	3.88	4	3.81	77/36	C
Psychological and social support focused on body image issues, mental well-being, and coping strategies can be important for managing the symptom burden in patients living with lipedema.^42,61-64^	Social and psychological aspects	4.59	4.54	4.6	4.45	76/37	C
Low-impact exercises, such as aquatic exercises, walking, and yoga, can help maintain mobility and support weight management in individuals with lipedema.^54,55^	Treatment	4.53	4.49	4.5	4.37	76/37	C
When lipedema coexists with obesity and metabolic disease, bariatric surgery should be prioritized before liposuction.^10,21,65-68^	Prevention/prognosis	3.99	3.99	4.05	3.84	75/38	C
Liposuction with preservation of lymphatics should be considered when it could significantly improve physical well-being.^21,67,68^	Treatment	4.12	4.11	4.15	4	76/37	C
Surgical interventions should be performed at experienced centers with proven expertise in the management of lipedema, including conservative treatment, as part of a comprehensive approach.^10,12,67^	Treatment	4.42	4.41	4.45	4.27	76/37	C
Increasing awareness of lipedema among the medical community and society is essential to reduce misdiagnosis and stigma.^12,14^	Multidisciplinary approach	4.62	4.64	4.67	4.5	76/37	C
Further research is needed to elucidate the underlying biological mechanisms of lipedema, leading to the development of objective diagnostic criteria and targeted therapies.^69^	Research and development	4.66	4.65	4.63	4.55	76/37	C
Long-term studies are needed to assess the efficacy and safety of treatment modalities for lipedema. In particular, surgical treatment should be investigated for its metabolic impact in the short, medium, and long term. The psychological impact should also be investigated.^12,55,69^	Research and development	4.68	4.64	4.61	4.6	76/37	C
Collaborative efforts among patients, researchers, clinicians, and support groups are essential to advance knowledge.	Research and development	4.68	4.69	4.72	4.65	76/37	C
Anti-inflammatory and ketogenic diets have shown symptomatic improvement in certain patients.^19,51^	Treatment	4.16	4.13	4.19	3.89	76/37	C
Lipedema symptoms may be exacerbated by prolonged periods of inactivity or air travel.^23,62^	Clinical characteristics	4.04	4.08	4.03	3.75	76/37	C
Increased adipose tissue in the arms and legs of patients with lipedema can hinder the performance of activities of daily living.^12,54,55^	Clinical characteristics	4.55	4.53	4.5	4.22	75/38	C
Patients with lipedema often report increased sensitivity in the affected areas.^10,12,41^	Clinical characteristics	4.45	4.41	4.47	4.26	73/40	B
Patients with lipedema may struggle to find clothes that fit properly due to increased limb size.^70-73^	Social and psychological aspects	4.58	4.4	4.54	4.22	73/40	C
Lipedema pain may interfere with sleep and adequate rest.^4,74-77^	Clinical characteristics	4.19	4.13	4.24	3.9	72/41	C
Early identification and adequate treatment of lipedema can help minimize disease progression.^12,13^	Prevention/prognosis	4.45	4.44	4.42	4.29	73/40	C
Self-awareness and patient education about lipedema are essential for proactive care and management at home.^10,61,78^	Social and psychological aspects	4.58	4.49	4.48	4.36	73/40	C
Manual lymph massage can help reduce swelling and pain associated with lipedema.	Treatment	4.26	4.21	4.21	3.99	73/40	C
Individuals with lipedema are at a higher risk of developing knee disorders such as chondromalacia and early osteoarthritis.^4,9,25^	Clinical characteristics	4.11	4.06	4.09	3.91	71/42	C
Joining an exercise program with a certified lymphedema therapists is crucial for preventing injuries.^54,55^	Multidisciplinary approach	4.44	4.31	4.39	4.1	68/45	C
The recommendation for surgery should be approved by the clinician, the surgeon, and the patient. It should prioritize improving mobility first and symptomatic relief second. Aesthetic outcomes should not be the main goal of surgical treatment for lipedema.^10^	Treatment	4.09	4.13	4.23	3.94	65/48	C
When lipedema coexists with obesity, its symptoms are expected to persist even after bariatric surgery.^12,40,66^	Prognosis	4.38	4.35	4.38	4.1	61/52	

Level of evidence A: Randomized clinical trials/meta-analyses; B: Case-control studies/single clinical trial/cohort studies; C: Case reports/expert consensus.

### Phase 3

The final phase of the consensus statement included 59 specialists. During this phase, 42 statements were evaluated with the aim of establishing a robust consensus on lipedema.

Of the 42 statements evaluated (Table 2),^4,5,9,10,12,13,20,21,26,27,29,30,32,34,37-39,42,47,49,53,54,60-64,74,76-78,80-108^ all reached significant consensus across the following criteria: “Agreement with the statement,” “The statement should be included in the final report,” and “The statement is clear.” This indicates strong overall agreement among specialists regarding the content and relevance of these statements.

**Table 2:** Phase 3 results.

**Statement**	**Topic**	**Agreement**	**Inclusion**	**Clarity**	**Evidence**	**Responded/skipped**	**Level of evidence**
The prevalence of advanced lipedema is uncertain due to a lack of comprehensive studies and underdiagnosis. Many cases may be confused with obesity, lymphedema, or chronic venous disease. It is crucial to identify the condition early to prevent progression. More research is needed to understand its true extent and improve patient follow-up.^63^	Epidemiology	4.75	4.6	4.6	4.24	55/3	C
In lipedema, increased pain and sensitivity usually occur in areas with excess adipose tissue but may also extend to adjacent regions due to sensitive nerves or systemic inflammation. This can lead to misdiagnoses, such as fibromyalgia. Therefore, careful evaluation is crucial for accurately determining the distribution and cause of pain in these patients.^20,29,80^	Clinical characteristics	4.52	4.44	4.4	4	55/3	C
Lipolymphedema occurs when lymphedema arises as a complication of lipedema due to chronic inflammation. However, if lymphedema is caused by other factors, such as a sedentary lifestyle or being overweight, it is classified as a comorbidity rather than a stage of lipolymphedema.^29,47,81-83^	Clinical characteristics	3.95	3.87	4.15	3.69	55/3	B
Alterations in the microvascular system appear to play a role in the onset of lipedema and may be present in all stages of the condition.^12,30,84,85^	Pathogenesis/pathophysiology	3.85	3.87	3.96	3.57*	52/6	C
Sensitivity and pain can be exacerbated by factors such as severe joint strain due to increased weight or skin irritation due to friction or inflammation. The presence of comorbidities such as fibromyalgia can further exacerbate these manifestations.^20,29,62,80^	Clinical characteristics	4.37	4.33	4.31	3.94	52/6	C
Lipedema is not caused by obesity, nor is obesity caused by lipedema; they are distinct conditions with their own pathophysiological processes. However, obesity can exacerbate the symptoms of lipedema, and significant weight gain may act as a trigger for the onset of lipedema. It is important for individuals, regardless of their weight, to seek medical evaluation if they suspect they have the condition, as early treatment can be beneficial. More studies are needed to fully understand the relationship between the two conditions.^12^	Pathogenesis/pathophysiology	4.46	4.4	4.48	4	52/6	C
Lymph stasis may occur at any stage of lipedema but is more evident and frequent in more advanced stages.^34,39^	Pathogenesis/pathophysiology	4.36	4.36	4.43	4.12	50/8	C
Individuals with lipedema, particularly those who maintain a healthy body weight, may exhibit a metabolic profile comparable to that of the general population, including a lower risk of conditions such as hypercholesterolemia and potentially reduced risk of cardiovascular diseases.^4,9,86,87^	Epidemiology	4.02	4.02	4.1	3.76	50/8	C
Studies suggest that the incidence of hypothyroidism may be higher in patients with lipedema than in those without the condition.^4^	Research and development	3.76	3.74*	3.98	3.6*	50/8	C
Lipedema may be associated with connective tissue disorders, such as Ehlers-Danlos syndrome, particularly concerning variations in the severity of joint hypermobility.^26,37,88^	Pathogenesis	3.81	3.8	3.92	3.57*	49/9	C
Lipedema may be associated with muscle weakness, affecting the ability to gain muscle mass and overall muscle function in both the proximal and distal regions of the upper and lower limbs.^54,89,90^	Clinical characteristics	4	4.06	4.16	3.84	49/9	C
The psychological impact suffered by patients with lipedema appears to be a consequence of the symptoms of the disease rather than an initial cause. However, it is essential to consider mental health as a key part of lipedema management, as psychological status and physical symptoms may be closely linked.^4,42,61,64^	Social and psychological aspects	4.65	4.59	4.59	4.35	49/9	C
During routine clinical examinations, it is recommended to use more specific anthropometric measurements, such as the waist-to-height and waist-to-hip ratios, for a more accurate assessment. Additional methods, such as whole-body bone density scans (DXA) and bioimpedance analysis, may be considered for a more detailed characterization of body composition, as relying solely on body mass index may not accurately reflect the specific characteristics of lipedema.^60,91^	Diagnosis	4.63	4.53	4.61	4.35	49/9	C
The classification of lipedema into stages is based on the distribution and progression of adipose tissue and does not necessarily reflect the severity of symptoms, which can vary according to inflammation and associated comorbidities. While patients in advanced stages may report more symptoms, the experience of pain and other complaints is subjective and may not correlate directly with the visual stage of the disease.^10,12,92^	Classification	4.64	4.64	4.62	4.27	45/13	C
The clinical classification of lipedema into stages can influence the choice of treatment, with the need for surgical intervention depending on the stage of the condition. While early stages may not require surgery, more advanced stages – especially when there is significant mobility restriction – may require specific surgical approaches.^10,12,49,78,92^	Classification	4.4	4.42	4.56	4.56	45/13	C
The progression of lipedema symptoms can be alleviated or even prevented with adequate treatment and the adoption of healthy lifestyle habits.	Treatment	4.71	4.69	4.69	4.47	45/13	C
Lipedema is characterized by increased limb volume, which can lead to higher body weight. It can be differentiated from common obesity by the distribution of fat and its resistance to conventional weight loss methods.^9,12,93^	Clinical characteristics	4.51	4.5	4.58	4.31	45/13	C
Clinical classifications based on the location of lipedema are useful for characterizing fat distribution and can inform diagnosis and treatment planning, while also indicating potential progressions and associated complications.^10,12,77^	Classification	4.29	4.24	4.24	3.93	45/13	C
Stress management techniques, such as mindfulness and breathing exercises, contribute to overall well-being and may improve adherence to lipedema treatment, in addition to helping manage the emotional impact and pain associated with the condition.^61,64^	Treatment	4.31	4.18	4.33	3.98	45/13	C
Valgus knees is commonly observed in individuals with lipedema, which can affect gait and increase the risk of complications such as gonarthrosis, directly impacting quality of life and mobility.^9,76^	Clinical characteristics	4.36	4.36	4.41	4	45/13	C
Individuals with lipedema often have a high intake of carbohydrates and potentially inflammatory foods, which may result in low protein intake relative to daily recommendations.^94-96^	Multidisciplinary approach	4.36	4.33	4.49	4.09	45/13	C
Symptomatic individuals with lipedema often experience food intolerances.^38^	Clinical characteristics	3.91	3.86	4.09	3.7*	45/13	C
Individuals with symptomatic lipedema tend to lead a sedentary lifestyle, although exercise, when practiced, should be carefully chosen to avoid high-impact activities that may worsen symptoms.^97,98^	Clinical characteristics	4.44	4.33	4.4	4.09	45/13	C
Individuals with symptomatic lipedema often experience respiratory allergies, such as rhinitis and sinusitis.^99^	Clinical characteristics	3.6	3.58*	3.88*	3.49*	45/13	C
Individuals with lipedema may experience changes in bowel habits, often as a result of a diet high in inflammatory foods and alterations in gut flora, which can contribute to a systemic inflammatory state.^95,100^	Clinical characteristics	4.05	4.05	4.23	3.84	45/13	C
Individuals with lipedema, especially in more advanced stages, often struggle to use elastic bandages and other compression garments due to significant limb disproportion and discomfort caused by compression, which can be exacerbated during periods of increased inflammation. These garments should be custom-made for each individual.^30,82,101^	Treatment	4.65	4.58	4.6	4.28	45/13	C
Individuals with symptomatic lipedema often report sleep problems.^4,74^	Clinical characteristics	3.81	3.67	4.05	3.56	45/13	C
Individuals with symptomatic lipedema often do not meet the daily recommended water intake.	Clinical characteristics	4.02	3.98	4.23	3.79	43/15	C
Individuals with symptomatic lipedema often have fiber intake below the recommended daily amount.	Clinical characteristics	4.17	4.05	4.31	3.88	42/16	C
Although a relationship between lipedema and attention deficit hyperactivity disorder has been proposed, it still requires further research. Individuals with lipedema often exhibit impulsivity and require psychological support. Surgery is only indicated when impulsivity is under control. Additionally, lipedema may be associated with other mental health disorders.^61^	Social and psychological aspects	3.93	3.9	4.1	3.64	42/16	C
Young patients in the early stages of lipedema tend to have good postoperative outcomes; however, conservative treatment is beneficial for all individuals. Surgical indications and their effects on functionality, as well as aesthetic considerations, should always be taken into account. The impact on fertility remains uncertain.^5,53,77^	Treatment	3.95	3.95	3.9	3.67*	42/16	C
The decision to perform surgery for the treatment of lipedema should not be made hastily. It should only be considered after 1 year of clinical treatment, ensuring that the patient understands their condition. However, depending on the severity of the disease and the patient’s response to clinical treatment, surgery may be beneficial in advanced stages to improve mobility.^5,10,102^	Treatment	4.46	4.45	4.49	4.27	41/17	C
The progression of lipedema occurs during episodes of inflammation, during which the disease advances. In the absence of inflammation, the condition remains stable.^21,32^	Treatment	4.08	3.9	4.15	3.75	40/18	C
The diagnosis of lipedema typically involves an initial assessment that includes ultrasound to rule out edema, Doppler ultrasound to exclude varicose veins, and photoplethysmography to assess venous function. If these tests do not reveal any vascular issues, discharge from a venous perspective may be considered.^27,103,104^	Diagnosis	3.63	3.63	3.95	3.39*	38/20	C
Vitamin D deficiency is common among women with lipedema, highlighting the importance of monitoring and adequate supplementation of this vitamin.^105,106^	Treatment	4.13	4.15	4.28	3.92	39/19	C
Muscle-strengthening exercises, particularly those targeting the hamstrings, have been shown to be beneficial for women with lipedema, contributing to improved quality of life and symptom management.^54,90^	Treatment	4.29	4.28	4.41	4.05	39/19	C
The basal metabolic rate in patients with symptomatic lipedema tends to be lower than expected, which may influence management strategies and nutrition counseling for these patients.^86^	Multidisciplinary approach	4.03	3.97	4.18	3.79	39/19	C
Self-care in the treatment of lipedema can include the use of soft-bristled brushes for self-massage, vibration platforms, and elastic bandages.^10,107,108^	Treatment	4.23	4.15	4.26	3.95	39/19	C
Routine use of quality-of-life questionnaires is recommended to assess the efficacy of therapeutic interventions in the treatment of lipedema.^92^	Treatment	4.56	4.51	4.59	4.31	39/19	C
Evaluating triggers for lipedema exacerbation, such as dietary, hormonal, and social or professional stressors, can improve disease management.^21^	Diagnosis	4.67	4.59	4.67	4.44	39/19	C
A relationship has been observed between lipedema and varicose veins; however, this association requires further investigation. When treating associated venous disease, it is important to consider the presence of lipedema and establish a causal relationship between the symptoms.^4,12,13,101^	Research and development	4.23	4.18	4.26	4.05	39/19	C
Individuals with lipedema should not be encouraged to undergo multiple expensive treatments that have no proven efficacy.	Treatment	4.87	4.82	4.82	4.62	39/19	C

Level of evidence A: Randomized clinical trials/meta-analyses; B: Case-control studies/single clinical trial/cohort studies; C: Case reports/expert consensus.

However, 9 statements did not meet the required level of evidence for the criterion “There is sufficient evidence to support this statement.” This suggests that, although specialists agreed on the validity of these statements, there was a recognized need for further research or data to fully support these specific aspects of lipedema.

No new suggestions for additional statements were made, implying that the proposed set of statements was considered comprehensive and satisfactory by the participants, adequately representing the current knowledge of lipedema.

This consensus statement represents a significant step in consolidating the understanding of lipedema, laying a solid foundation for clinical practice and future guidelines, while also highlighting areas that require further investigation to strengthen the available evidence on lipedema treatment.

**Figure 4:**
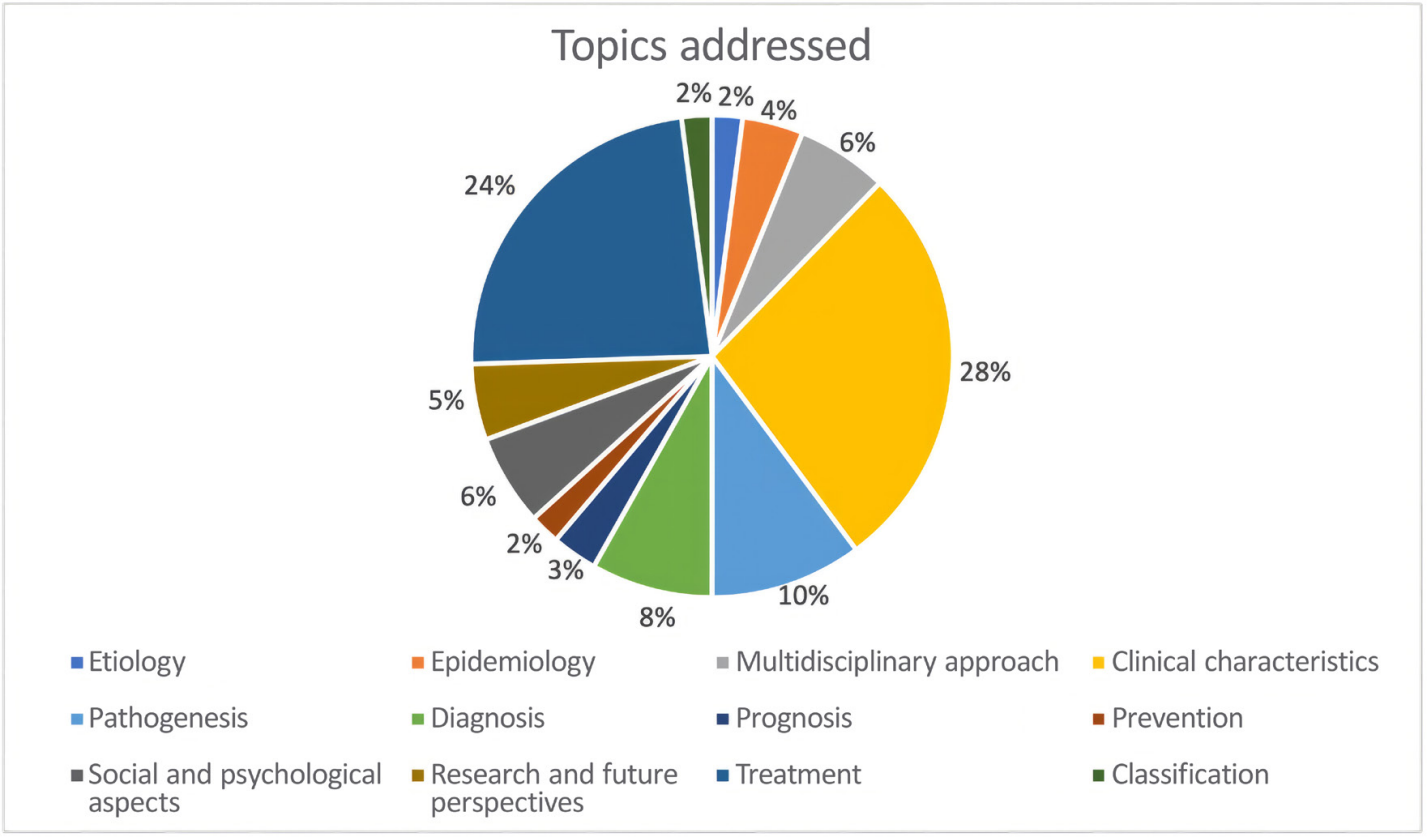
Topics addressed by the statements.

The results from Phase 2 are described in Table 2, and the topics addressed are shown in Figure 4.

## DISCUSSION

This Brazilian Consensus Statement on Lipedema represents a significant collaborative effort to outline the characteristics, diagnosis, and treatment of this complex condition. Because lipedema is a multifaceted condition that interacts with multiple body systems and has a heterogenous presentation among patients, reaching consensus is particularly challenging. As a chronic disease with no known cure, lipedema management requires a multidisciplinary approach, involving experts from several health fields.

The statements included in this document highlight the multidimensionality of lipedema. By affecting multiple body systems, lipedema affects the mental health, physical function, and quality of life of patients. It should not be reduced to a “fat disorder” or confused with other comorbidities, as it presents unique and distinct characteristics. The need for further research is evident to fully understand the pathophysiology of lipedema, especially concerning its mechanisms and optimal treatment practices.

### Epidemiology and clinical characteristics

The prevalence of lipedema in Brazil, reported at 12.3% of the adult female population,^4^ underscores the public health relevance of this condition and the need for increased awareness among physicians. This rate suggests that lipedema is common; however, it is often underdiagnosed or mistaken for other conditions, such as obesity. The overlap with obesity is frequent and can complicate both the diagnosis and treatment of lipedema, as the two disorders can coexist and interact, affecting the overall health of the patient.^4,12,23^

The uncertainty regarding the prevalence of advanced lipedema exposes a significant gap in current medical knowledge and highlights the need for further epidemiological research. Differentiating between lipedema and other similar conditions, such as lymphedema and chronic venous diseases, is challenging due to overlapping symptoms and a lack of awareness about lipedema. This underscores the importance of more refined diagnostic approaches and implementing assessment protocols that effectively distinguish between these conditions.^63^

Early detection of lipedema is crucial to prevent progression to more advanced stages, which are more difficult to manage and significantly impact quality of life and mobility. Early identification and treatment can help mitigate the development of complications and functional decline.^12^

Interestingly, some individuals with lipedema who maintain a healthy weight exhibit a metabolic profile comparable to the general population. This challenges many misconceptions about lipedema, suggesting that lipedema alone, without coexisting conditions such as obesity, may not be associated with an increased risk of metabolic comorbidities.^4,9,86,87^ However, it is important to note that lipedema can affect exercise capacity and lifestyle, which are key factors in maintaining a healthy metabolic profile.^97,98^

The expert panel agreed that lipedema presents particular clinical characteristics that need to be recognized. Lipedema is characterized by a symmetrical and bilateral increase in subcutaneous adipose tissue in the legs and arms, typically sparing the hands and feet.^10^ Fat buildup in lipedema is not merely an aesthetic issue but a painful and tender condition that can be severely debilitating.^14,16^ Patients often experience pain and a sensation of heaviness in the affected areas, along with a tendency to bruise easily, which may indicate connective tissue disorders or vascular fragility.^17,18^

Although lipedema predominantly affects individuals assigned female at birth and is closely linked to hormonal fluctuations during puberty, pregnancy, and menopause, rare cases in men have been observed. These hormonal events may act as triggers for the onset or worsening of lipedema symptoms.^9,12,30,31^

Lipedema can be exacerbated by periods of inactivity or prolonged air travel, suggesting that regular movement is beneficial in managing symptoms. Furthermore, increased adipose tissue can make it difficult to perform activities of daily living and even choose appropriate clothing, contributing to psychological stress and body image issues.^70-73^ Patients may also report increased sensitivity in the affected areas, with pain significantly interfering with everyday living and sleep quality.^20,29,80^ Thus, lipedema is not merely a physical condition; it also significantly impacts the mental well-being and quality of life of patients. Self-awareness and education about the disease are essential for patients to adopt proactive self-care and management strategies to improve their quality of life.^10,61,78^

The clinical presentation of lipedema is complex and multifaceted, with several manifestations that can significantly impact the well-being of patients. Pain and increased sensitivity are key features of lipedema, often located in areas with excess adipose tissue.^20,29,80^ These symptoms may be confused with other conditions, such as fibromyalgia, due to the similar distribution of pain. However, distinguishing between these two conditions is crucial for ensuring effective management.^20,29,62,80^

Lipolymphedema occurs when lymphedema arises as a complication of lipedema due to chronic inflammation. Identifying the underlying cause of lymphedema is crucial, as it can significantly impact treatment options and long-term outcomes.^29,47,81-83^ The pain associated with lipedema may be exacerbated by joint strain due to increased weight or skin irritation due to friction or inflammation.^9,76^ The presence of comorbidities, such as fibromyalgia, may further aggravate these symptoms.^20,29,80^

Patients with lipedema may present muscle weakness, which affects both muscle function and the ability to gain muscle mass. This can compromise mobility and the ability to perform activities of daily living, increasing the risk of sedentary behavior.^54,89,90^ The atypical fat distribution in lipedema differs from conventional obesity and is often resistant to traditional weight loss methods such as diet and exercise, leading to a cycle of weight gain and frustration for the patient.^9,12,93^

Orthopedic deformities, such as valgus knees, are common and can affect gait, increasing the risk of orthopedic complications such as osteoarthritis and further affecting quality of life and mobility.^9,76^ Additionally, food intolerance and changes in bowel habits may indicate a systemic inflammatory response that exacerbates lipedema symptoms. Respiratory allergies and sleep disorders are also frequently reported and may be related to the chronic inflammatory state. Low water and fiber intake can contribute to bowel problems and poor overall disease control. It is essential for patients with lipedema to maintain a balanced diet and meet daily nutrient intake recommendations to help mitigate these effects.

Recognizing and understanding these clinical aspects is crucial for health care professionals to provide comprehensive and individualized support to patients with lipedema, aiming not only to manage symptoms but also to improve their overall quality of life.

### Social and psychological aspects

The impact of lipedema on mental health and quality of life cannot be underestimated. This chronic physical disorder often leads to mental health issues, including depression, anxiety, and low self-esteem, exacerbated by chronic pain and physical limitations.^4^ These factors may lead to a vicious cycle of declining mental health and quality of life, highlighting the role of psychological interventions and social support as integral parts of lipedema management. Most statements on this topic were readily approved during Phase 2 of the consensus.

The hypothesis that lipedema may be correlated with attention deficit hyperactivity disorder (ADHD) is intriguing and suggests a potential complex neuropsychiatric condition underlying lipedema.^61^ Impulsivity, a common symptom in individuals with ADHD, may also manifest in patients with lipedema, impacting disease management and decision-making about treatments, including surgery. Adequate management of impulsivity is essential prior to any surgical procedure to ensure that patients’ decisions align with their desired outcomes and that they are prepared for postoperative care. Additionally, the association between lipedema and other psychiatric disorders underscores the importance of integrating psychological evaluation and support into the treatment plan.^42,61-64^

Psychological and social support is essential in addressing the psychosocial challenges imposed by lipedema. Body image issues and the stigma associated with weight gain can be devastating. Coping strategies, including cognitive-behavioral therapy and support groups, can be crucial for helping patients deal with the emotional and symptomatic burden of lipedema, promoting mental well-being and facilitating adaptation to the disease.^42,61-64^

Patients with lipedema often struggle to find clothes that fit properly, leading to additional stress and low self-esteem. This situation reveals the need for thoughtful ergonomic design in fashion, as well as the need for guidance on adaptations or custom-made garments that can improve comfort and promote body acceptance.^70-73^

Self-awareness and patient education are critical components of effective self-care. Knowledge of the disease allows patients to recognize symptoms early, seek adequate care, and adopt home management practices that can prevent disease progression and improve quality of life. Education also empowers patients to advocate for their own health care needs and seek appropriate support and resources.

### Classification

Lipedema is classified based on anatomical location and stage. Statements about classification had high agreement levels in Phase 3 of this study. Staging lipedema provides a useful framework for understanding disease progression, but may not accurately represent the heterogeneity of symptoms between patients. For example, two patients in the same disease stage may report very different levels of pain and disability.^21^ This highlights the importance of a holistic approach that considers not only the physical appearance of the patient but also the presence of comorbidities, inflammatory state, and psychosocial impacts.^10,12,92^

Classifying lipedema based on its location helps health care professionals to determine the extent of disease and plan treatment. However, this approach may sometimes oversimplify the complexity of individual cases by not accounting for the full spectrum of lipedema manifestations, such as pain and quality of life. Thus, while clinical classifications help identify and manage lipedema, they should be used alongside a detailed clinical evaluation.^10,12,49,78,92^

The stage of lipedema directly affects the type of treatment, which can range from conservative management to surgery. Surgery, such as liposuction, is more commonly indicated in the advanced stages of the disease, when mobility and quality of life are significantly impaired. However, the decision to perform surgery should be carefully considered and based on a comprehensive individual assessment, including the patient’s wishes and expectations, as well as the risks and benefits.^10,12,77^

In summary, although staging is a valuable part of lipedema management, the complexity of the disease requires a personalized approach that considers the patient’s subjective experience and the overall impact of the disease on their life.

### Etiology, pathogenesis, and pathophysiology

Lipedema is a chronic, systemic disease, meaning it is long-lasting and affects multiple organ systems. Because of its chronic nature, lipedema requires ongoing management and may progress to advanced stages, often leading to increased fat buildup and associated symptoms such as pain, tenderness, and swelling.^21,32^ In addition, although symptoms are most noticeable in the lower limbs, the effects of lipedema may be more widespread, potentially impacting the circulatory and lymphatic systems and contributing to chronic inflammation throughout the body.^40,82,98,109,110^

Furthermore, evidence suggests a hereditary component to lipedema. Several studies indicate a genetic predisposition, as the condition tends to appear in families, suggesting a pattern of inheritance. However, the exact genetics of lipedema are not fully understood and are believed to be polygenic, ie, involving the contribution of multiple genes. Environmental factors and lifestyle habits may also influence the manifestation and severity of lipedema.^25,111^

Recognizing lipedema as a chronic, systemic disease with a potential hereditary origin is crucial for developing effective treatment strategies and providing adequate patient support.^9,10,25^ Ongoing research is also necessary to better understand the pathogenesis of the condition and to develop personalized treatment approaches based on genetics and underlying biological mechanisms.

The pathogenesis of lipedema is multifactorial, with a potential link to connective tissue abnormalities, as evidenced by its association with Ehlers-Danlos syndrome and joint hypermobility.^26,37,88^ Lipedema is characterized by an atypical accumulation of adipose tissue in the limbs, sparing the trunk and often affecting the upper limbs symmetrically and bilaterally.^10^

Inflammation, hormonal changes, and increased extracellular fluid in adipose tissue have been identified as contributing factors to the pathogenesis of lipedema. Additionally, microvascular dysfunction may play a central role in the development of the condition.^84,112,113^ Although lipedema is not caused by obesity, and vice versa, obesity can exacerbate lipedema symptoms and may even trigger its onset in predisposed individuals. Lymph stasis, or lymph stagnation, can occur at any stage of lipedema, but it tends to be more prevalent in advanced stages.^19,47,82,83,109,110^

The full understanding of these interactions and the development of effective treatment strategies are crucial. It is essential to recognize that while lipedema and obesity may coexist, they have distinct etiologies and thus require different diagnostic and management approaches.

### Multidisciplinary approach

Lipedema is a chronic and progressive adipose tissue disorder that is poorly understood and often underdiagnosed. It is characterized by a symmetrical buildup of fat in the limbs, typically sparing the hands and feet, and may be accompanied by pain and edema. The pathogenesis of lipedema is not fully understood but it is believed to involve genetic, hormonal, and inflammatory factors. Given its complex and multifaceted nature, lipedema treatment requires a multidisciplinary approach, addressing not only the physical symptoms but also providing psychological support. Therefore, statements recommending multidisciplinary approaches were strongly supported during Phase 2 of this consensus statement.

A multidisciplinary approach is essential for the treatment of lipedema, as specialists from different fields can contribute their expertise to create a comprehensive treatment plan. Vascular surgeons can manage associated lymphatic complications, while endocrinologists can help optimize the hormonal and metabolic profiles of patients. Orthopedists can treat musculoskeletal disorders resulting from increased limb weight, and plastic surgeons can perform procedures to reduce fat buildup, improving both functionality and aesthetics. Psychiatrists and mental health professionals play a key role in addressing emotional and psychological problems, which are often overlooked. Additionally, gynecologists may contribute to managing hormonal imbalances and gynecological problems, especially in women with lipedema.^48-50^

Raising awareness about lipedema is crucial to improve the recognition and treatment of the condition. Many patients go years without a diagnosis or may be misdiagnosed with, for example, obesity or lymphedema, often leading to ineffective treatments and increased stigma and frustration. Therefore, continuous education for health care professionals and raising public awareness are essential steps to reduce these challenges and promote a more effective and compassionate therapeutic approach.^10,61,78^

Physical exercise is an important component in the management of lipedema, as it helps to improve mobility, reduce the risk of complications such as lymphedema, and maintain cardiovascular health. However, exercises should be tailored to each individual, considering pain and mobility limitations. Certified lymphedema therapists can develop exercise programs that minimize the risk of injury while maximizing therapeutic benefits, considering the specific needs and limitations of each patient.^54,55^

The topic of dietary approaches, especially regarding the balance between carbohydrates and proteins, was discussed in Phase 3 of this consensus statement. Although somewhat more controversial, statements about diet had high agreement levels and were supported by strong evidence. It is essential for nutritionists, dietitians, endocrinologists, and all professionals involved in lipedema care to have access to specialized nutrition training and to establish a unified approach with both the health care team and the patient. Disagreements among providers may hinder patients’ adherence to dietary plans, which is already challenging.^86^

### Treatment

Lipedema treatment should involve a multidisciplinary approach that covers nutrition, exercise, clinical care, and surgery when necessary. Although some strategies remain controversial, it is widely accepted that noninvasive or conservative treatment should be the initial step and is an essential part of disease management. Surgery should not precede conservative clinical treatment, which remains fundamental even after surgical procedures.

### Conservative treatment

The main goal of conservative treatment in lipedema is to improve the patient’s quality of life by alleviating symptoms and slowing disease progression. Adequate management involves a holistic and multidisciplinary approach focused on lifestyle changes, such as a balanced diet and regular low-impact exercise, to help reduce pain and sensitivity. Compression therapy and physical therapy, such as complex decongestive therapy (CDT), have been shown to be effective in reducing discomfort and edema, although individual variation in treatment response is common.^30,82,101^

Furthermore, weight control is essential, especially when lipedema is accompanied by obesity, which can worsen inflammatory symptoms. Strategies to achieve a healthy weight should focus on anthropometric measurements, such as the waist-to-height ratio, to assess the risks associated with excess body fat. Slowing the progression of lipedema symptoms can be achieved through a healthy lifestyle and adequate treatment, including measures to prevent inflammatory episodes that may accelerate the condition.^59,60^

To monitor the effectiveness of therapeutic interventions, the use of quality of life assessment tools, such as the QuASiL and 36-Item Short Form Survey, is recommended. These tools allow for continuous and objective evaluation of the therapeutic impact on patient well-being, allowing adjustments to the treatment plan when necessary to optimize outcomes.^92^

Finally, it is crucial that proposed therapies be based on solid scientific evidence. Patients should not be encouraged to undergo multiple expensive treatments that have no proven effectiveness. The integrity of lipedema treatment relies on the judicious, evidence-based use of therapeutic resources, with the sole objective of providing the best possible care for patients.

### Compression therapy and physical exercise

Within the therapeutic spectrum for lipedema, manual lymph massage emerges as a valuable technique for alleviating swelling and pain. This technique improves circulation and lymphatic flow, providing both physical and psychological benefits for patients.^30,82,101^

CDT is a cornerstone of lipedema treatment, even in early stages. By comprising strategies such as skin care, manual lymphatic drainage, exercises, and compression, CDT effectively controls the progression of lipedema by preventing fluid buildup and swelling progression.^56-58^

Low-impact exercises, such as aquatic exercises, walking, and yoga, are recommended to help maintain mobility and for weight management in patients with lipedema. These exercises are gentle on the joints, which is particularly beneficial given that lipedema can cause joint pain and fragility. Muscle-strengthening exercises, particularly those targeting the hamstrings, have been shown to effectively manage lipedema symptoms and improve patient function and quality of life.^54,55^

As lipedema progresses, adherence to compression therapies, such as elastic bandages, may become more challenging due to disproportionate limb size and potential discomfort. Thus, custom-made compression garments are essential to ensure treatment efficacy and patient comfort, especially during episodes of pronounced inflammation.^30,82,101^

Self-care is fundamental to lipedema management. Techniques such as self-massage with soft-bristle brushes, vibration plates, and consistent use of compression bandages can provide autonomy for patients, giving them additional control over symptoms and enhancing the benefits of clinical treatments.^10,107,108^

### Psychological and nutrition counseling

Stress management techniques, such as mindfulness and breathing exercises, are known to improve overall well-being. In patients with lipedema, these methods can be particularly helpful in alleviating psychological stress, reducing chronic pain, and improving treatment adherence – aspects that are often challenging for these patients.^61,64^

Nutrition counseling is a cornerstone of lipedema management, directly impacting patients’ ability to manage their weight and optimize their overall health. A balanced diet can help reduce systemic inflammation and lipedema-related symptoms, potentially improving the efficacy of other therapeutic interventions. Specific dietary approaches, such as anti-inflammatory and ketogenic diets, have been investigated in patients with lipedema and may help reduce symptoms. However, these dietary strategies should be carefully evaluated and tailored to individual needs, considering the variability in treatment response among patients.^51,52,95,100,114-117^

Vitamin D is a nutrient with multiple biological functions, including modulation of immune and inflammatory responses, and is frequently deficient in women with lipedema. Therefore, adequate monitoring and supplementation are essential for bone health and possibly slowing lipedema progression. The maintenance of adequate vitamin D levels may help alleviate symptoms and prevent complications associated with lipedema.^105,106^

### Surgical intervention

Surgical interventions for lipedema should be considered within a comprehensive care spectrum and performed at specialized centers with expertise in both conservative and surgical treatments. The experience of the medical center is essential to ensure a holistic and individualized approach to lipedema treatment.

The decision for surgical intervention should be a collaborative process involving the clinician, surgeon, and patient. Functional improvement, particularly in mobility, should be the primary goal, followed by symptom relief. Surgical decisions should not be made hastily, especially in light of the dearth of large studies with long-term follow-up that examine all aspects of lipedema. While aesthetic outcomes may be a secondary benefit, they should not be the main focus.^5,10,102^

Younger patients in early stages of lipedema often experience positive surgical outcomes; however, these are also the patients who tend to benefit the most from conservative treatments. Conservative treatment offers universal benefits and should be prioritized over surgery, which requires careful consideration of functional impacts and potential effects on fertility, which remain uncertain.^5,19,53,77^

Surgery as a treatment for lipedema should be considered only after careful evaluation. Typically, it is recommended to fully explore clinical treatment options for at least 1 year before considering surgery. A patient’s understanding of their condition and their response to clinical treatment are essential components of this decision-making process. However, in cases of advanced lipedema where mobility is significantly impaired, surgical intervention may be considered earlier.^5,10,102^

### Prevention and prognosis

At the moment, it is difficult to establish a clear prognosis or definitive preventive measures for lipedema. However, some recommendations are widely agreed upon, as evidenced by the prompt acceptance of all statements during Phase 2 of this study.

When lipedema coexists with obesity and metabolic diseases, weight management through bariatric surgery may be considered an initial step before specific lipedema treatments such as liposuction. This is because weight reduction can help relieve pressure on the lymphatic system and mitigate lipedema-related symptoms.^10,21,65-68^

Early identification and intervention are crucial for preventing disease progression and improving prognosis.^21^ Early treatment can stabilize fat buildup and prevent complications such as lymph stasis and fibrosis.

Bariatric surgery may lead to significant weight loss in patients with obesity. However, it should be noted that lipedema symptoms, such as characteristic fat buildup and pain, may not resolve solely through weight loss and may require additional treatment.^25,40,41^

Delayed diagnosis and treatment of lipedema may increase the symptom burden and negatively impact the mental health and quality of life of patients. Disease progression to more advanced stages, which are harder to treat, can increase disability and pain.

### Future perspectives, research, and development

Although lipedema was first clinically described in 1940, only recently has there been a surge in publications on the condition. Further research to clarify the biological mechanisms underlying lipedema is essential. A deeper understanding of the disease’s etiology is crucial for the development of accurate diagnostic criteria and more effective and personalized therapies. This is particularly important given the complexity of lipedema symptoms and the variability in patient response to treatment.

Long-term studies are indispensable, not only to assess the safety and efficacy of therapeutic interventions but also to understand the long-term effects of these treatments, especially surgical ones, which may have significant physical, metabolic, and psychological impacts. Long-term follow-up will help elucidate the disease’s course and the sustained efficacy of different treatment modalities.

Collaboration among patients, researchers, clinicians, and support groups is crucial for advancing knowledge of lipedema. These partnerships can improve the design and implementation of studies, ensure that patient perspectives are considered, and expedite the clinical application of findings.^69^

The possible link between lipedema and endocrine conditions such as hypothyroidism suggests the existence of common underlying systemic mechanisms that require further investigation. This could lead to a better understanding of how lipedema affects and is influenced by other systemic conditions.^12,55,69^

Finally, the association between lipedema and varicose veins highlights the importance of considering the interaction between multiple vascular conditions. This may have implications for treatment, suggesting an integrated approach to management.

This consensus statement represents a significant advancement in understanding lipedema in Brazil, marking the beginning of a journey toward clearer insights and improved practices in diagnosing and treating lipedema. We recognize that there is a long and challenging path ahead, but this joint effort provides a solid foundation for future research and improved patient care.

## CONCLUSION

The first Brazilian Consensus Statement on Lipedema underscores the complexity of this condition and the necessity of a multidisciplinary treatment approach. While we have reached significant consensus, there remains an absence of absolute consensus and a standardized treatment protocol. Conservative treatments should be prioritized, with surgery considered only after all options have been exhausted. This consensus statement is a crucial step forward in improving the diagnosis and treatment of lipedema; however, we recognize the ongoing need for research and the development of more effective disease management strategies.
