# Supplementary material for: Brazilian Consensus Statement on Lipedema using the Delphi methodology
Source: J Vasc Bras. 2025 Feb 10;24:e20230183. doi: 10.1590/1677-5449.202301832 (PMC11815829; doi:10.1590/1677-5449.202301832)
Supplement: Translation

# Translation: Consenso Brasileiro de Lipedema pela metodologia Delphi

**DOI:** 10.1590/1677-5449.202301831

**Authors:** Alexandre Campos Moraes Amato, Ana Paula Rolim Maia Peclat, Rodrigo Kikuchi, Antonio Carlos de Souza, Mariana Thalyta Bertolin Silva, Roney Hans Prager de Oliveira, Daniel Augusto Benitti, Julio Cesar Peclat de Oliveira

**Affiliations:** 1 Sociedade Brasileira de Angiologia e de Cirurgia Vascular – SBACV, São Paulo, SP, Brasil.; 2 Amato – Instituto de Medicina Avançada, São Paulo, SP, Brasil.; 3 Faculdade Souza Marques – FTESM, Rio de Janeiro, RJ, Brasil.; 4 Clínica Peclat, São Gonçalo, RJ, Brasil.; 5 Santa Casa de São Paulo, São Paulo, SP, Brasil.; 6 Instituto de Excelência Vascular, Londrina, PR, Brasil.

**Keywords:** lipedema, obesidade, linfedema, consenso

## Abstract

O lipedema, historicamente sub-reconhecido, tem ganhado destaque devido aos avanços na pesquisa e ao aumento da conscientização. O Consenso Brasileiro de Lipedema, promovido pela Sociedade Brasileira de Angiologia e de Cirurgia Vascular, visa estabelecer diretrizes claras para diagnóstico, tratamento e manejo. Utilizando a metodologia Delphi, o estudo envolveu a criação de 90 afirmações sobre lipedema por especialistas, seguida pela avaliação de 113 profissionais adicionais. Essas afirmações foram analisadas via SurveyMonkey, com um limiar de 75% de concordância necessário para sua inclusão no consenso. A maioria das afirmações atingiu um consenso significativo, exceto nove, que precisam de mais pesquisa. O estudo enfatiza a complexidade do lipedema, a eficácia do tratamento conservador sobre a cirurgia, a necessidade de abordagens multidisciplinares e a importância da conscientização para reduzir o subdiagnóstico e o estigma. Ressalta-se também a contínua necessidade de pesquisas para desenvolver estratégias de manejo mais efetivas.

## INTRODUÇÃO

O lipedema, uma condição clínica reconhecida há décadas^1^ , passou por um período de obscuridade e mal-entendidos, apesar de o Brasil ter sido pioneiro no tratamento cirúrgico, com o Prof. Ivo Pitanguy^2^ . Essa condição permaneceu relativamente obscura tanto na literatura médica internacional quanto nacional^3^ , assim como na consciência pública. Recentemente, contudo, observou-se um ressurgimento do interesse pelo lipedema, impulsionado por avanços na pesquisa médica e novas técnicas cirúrgicas, além de uma crescente conscientização entre pacientes e profissionais de saúde. Esse interesse renovado sublinhou a necessidade de estabelecer diretrizes claras para a identificação, o manejo e o tratamento do lipedema. O objetivo deste trabalho é divulgar os resultados do Consenso Brasileiro de Lipedema da Sociedade Brasileira de Angiologia e de Cirurgia Vascular (SBACV), um esforço colaborativo e multidisciplinar para esclarecer dúvidas, estabelecer parâmetros clínicos e orientar a abordagem dessa condição no Brasil, buscando uma compreensão mais profunda e um tratamento mais eficaz para o lipedema.

O lipedema, reconhecido como uma doença complexa, afeta inúmeros aspectos da saúde feminina, desafiando os paradigmas clínicos e terapêuticos existentes^4^ . Essa complexidade tem contribuído para uma prática clínica fragmentada, em que a ausência de um consenso estabelecido leva ao surgimento de protocolos individuais e uma espécie de “corrida do ouro” terapêutica. Nesse cenário, profissionais da saúde buscam oferecer soluções baseadas em suas experiências pessoais e perspectivas individuais, muitas vezes sem um embasamento científico sólido ou consensual.

Este fenômeno é particularmente evidente na abordagem cirúrgica do lipedema. Os procedimentos cirúrgicos, frequentemente considerados como soluções potenciais, ainda carecem de validação rigorosa e consenso na comunidade médica^5^ . A falta de um entendimento completo e integrado da fisiopatologia do lipedema adiciona outra camada de complexidade, limitando a capacidade dos profissionais de saúde de oferecer tratamentos fundamentados e eficazes.

No entanto, apesar dessas incertezas e desafios, os pacientes com lipedema existem e enfrentam dificuldades diárias, demandando soluções urgentes^4^ . A necessidade de respostas para seus problemas atuais é uma realidade palpável e inadiável. Nesse contexto, a elaboração de um consenso não é apenas desejável, mas essencial. Um consenso proporcionaria um denominador comum, uma base sobre a qual protocolos de diagnóstico, tratamento e manejo possam ser construídos. Isso não só orientaria os profissionais de saúde em suas práticas clínicas, mas também ofereceria aos pacientes um caminho mais claro e seguro para o manejo de sua condição. Portanto, a necessidade de um consenso sobre o lipedema é uma questão premente, fundamental para avançar no cuidado e na compreensão dessa condição desafiadora.

A técnica Delphi, criada na década de 1950 na RAND Corporation por Norman C. Dalkey^6^ , é um método reconhecido para alcançar consenso entre especialistas em uma área específica. Essa técnica parte do princípio de que as opiniões coletivas de um grupo estruturado de especialistas são mais confiáveis do que as de grupos desorganizados ou indivíduos isolados. O método Delphi é notável por seu processo claro de coleta e análise de opiniões, mantendo o anonimato dos participantes (as identidades não são reveladas mesmo após a conclusão do estudo) e permitindo a inclusão de especialistas de diferentes regiões de forma econômica e eficiente^7^ .

Para evitar os problemas típicos de discussões em grupo, como a influência dos outros ou pressão para concordar com a maioria, as interações entre os participantes são limitadas^6^ . Os especialistas respondem a questionários em várias rodadas, e, após cada rodada, um facilitador fornece um resumo das respostas, sem identificar quem disse o quê^6-8^ . Isso permite que os participantes mudem suas opiniões nas rodadas seguintes, considerando as respostas médias do grupo. No final, são usadas análises estatísticas para uma avaliação objetiva e imparcial dos dados, assegurando que todas as opiniões sejam adequadamente representadas no resultado final do estudo^6-8^ .

O objetivo deste estudo foi desenvolver, na Fase 1, um conjunto de afirmações sobre o lipedema elaborado por um comitê de especialistas. Nas Fases 2 e 3, essas afirmações foram validadas e modificadas até que um consenso amplo fosse alcançado. Esse processo detalhado garantiu que as afirmações finais refletissem um acordo coletivo bem fundamentado entre os participantes, abordando a validade, clareza, relevância e fundamentação evidencial das afirmações.

## MÉTODOS

Todos os participantes deste estudo preencheram e assinaram o termo de consentimento. O estudo está em conformidade com os padrões estabelecidos pela Resolução 196/96 do Conselho Nacional de Saúde sobre pesquisa envolvendo seres humanos e com a Declaração de Helsinque.

### Fase 1 do estudo

Na primeira fase do nosso estudo de consenso sobre lipedema, sete autores, todos representando suas especialidades e membros da Comissão de Lipedema Biênio 2022/2023 da SBACV, com publicações e *expertise* comprovada no tema (pelo menos 8 anos), foram selecionados entre especialistas no assunto em grupos médicos e membros ativos da sociedade. Essa seleção refletiu a diversidade de conhecimentos e abordagens necessárias para uma compreensão abrangente do lipedema. Cada autor, distribuído pelo Brasil, trouxe sua experiência única para o projeto (Figura 1).

**Figura 1:** Fluxograma dos participantes e rodada da técnica Delphi. SBACV = Sociedade Brasileira de Angiologia e de Cirurgia Vascular.

O principal objetivo desta fase inicial era estabelecer as bases para o consenso. Para isso, os autores realizaram uma revisão detalhada das publicações existentes nas bases de dados MEDLINE, PubMed Central, Science Direct, Cochrane Library e Google Scholar sobre lipedema, trabalhando individualmente. Essa revisão teve como foco identificar os principais aspectos, desafios e lacunas de conhecimento relacionados ao lipedema.

Com base nesta revisão, os autores desenvolveram uma série de afirmações sobre o lipedema. Essas afirmações foram cuidadosamente elaboradas para cobrir os vários aspectos da doença, incluindo diagnóstico, tratamento e manejo. O intuito era que essas afirmações fossem posteriormente avaliadas nas fases seguintes do estudo quanto à sua clareza, relevância e embasamento científico.

Para facilitar a comunicação e a colaboração entre os autores, foi criado um grupo em uma plataforma de mensagens instantâneas. Esse espaço serviu como um fórum para discussões abertas, troca de ideias e refinamento das afirmações. É importante destacar que, nesta fase, não houve uma mensuração formal das opiniões de cada autor. O foco estava na discussão coletiva e na elaboração das afirmações, preparando o terreno para as avaliações mais estruturadas nas fases seguintes.

Dessa forma, a Fase 1 estabeleceu o alicerce para o estudo, garantindo que as afirmações subsequentemente avaliadas nas Fases 2 e 3 fossem abrangentes, pertinentes e refletissem uma compreensão multidisciplinar do lipedema. Os temas abordados foram etiologia, epidemiologia, abordagem multidisciplinar, características clínicas, patogênese/fisiopatologia, diagnóstico, prognóstico, aspectos sociais e psicológicos, tratamento, prevenção, pesquisa e desenvolvimento e classificação.

### Fase 2 do estudo

Na segunda fase do nosso estudo de consenso sobre lipedema, expandimos a participação para incluir 113 especialistas convidados, oriundos de todo o Brasil e representando uma ampla gama de especialidades médicas e áreas de saúde correlatas. Eles foram convidados por meio de mensagens oficiais da SBACV e grupos especializados em estudos do lipedema. Diferentemente da primeira fase, que envolveu um grupo menor e mais especializado, nesta fase optamos por não filtrar os participantes pela experiência específica no assunto. No entanto, foi solicitado que apenas aqueles que atuavam ativamente na área do lipedema participassem.

Utilizando a plataforma on-line SurveyMonkey (SurveyMonkey^®^, San Mateo, CA, EUA), foi realizada uma avaliação detalhada das 90 afirmações criadas na Fase 1. Os especialistas foram convocados por e-mail oficial da SBACV, incluindo membros de grupos de discussão sobre lipedema e participantes da SBACV. Cada participante revisou as afirmações individualmente e votou nelas com base em quatro critérios: concordância com a afirmação, se a afirmação deveria ser incluída no texto final, clareza da afirmação e se existia evidência suficiente para sustentar a afirmação.

Embora tenha sido necessário o preenchimento de dados de identificação por parte dos participantes, todas as opiniões sobre as afirmações foram anonimizadas, garantindo a objetividade e a imparcialidade do processo. Não houve interação direta entre os participantes, assegurando que as opiniões fossem independentes e não influenciadas por discussões grupais. Além disso, os especialistas tiveram a oportunidade de fornecer comentários qualitativos sobre cada afirmação e a possibilidade de sugerir novas afirmações para serem incluídas na próxima fase do estudo.

Essa abordagem permitiu uma ampla coleta de dados e perspectivas sobre as afirmações relacionadas ao lipedema, contribuindo para um consenso mais robusto e representativo das diversas visões e experiências dos profissionais envolvidos no diagnóstico e no tratamento dessa condição.

### Fase 3 do estudo

Na terceira fase do nosso estudo de consenso sobre lipedema, todos os especialistas que participaram das fases anteriores foram reconvocados, ressaltando a importância da continuidade de sua participação. Dessa convocação, 59 especialistas (51 cirurgiões vasculares, 3 angiologistas, 1 cirurgião plástico, 2 fisioterapeutas, 1 ginecologista e 1 nutricionista) participaram ativamente da etapa final.

As estatísticas da Fase 2 foram compiladas de forma anônima, e as afirmações foram reavaliadas e reelaboradas com base em quatro critérios estabelecidos:

1. **“Concordância com a afirmação”**: Afirmações com mais de 75% de concordância foram aceitas como parte do Consenso.
2. **“A afirmação deve ser incluída no texto final”**: Aquelas com mais de 75% de concordância foram aceitas para inclusão no documento de Consenso.
3. **“A afirmação está clara”**: Afirmações com menos de 75% de concordância foram revisadas e reescritas para nova validação, levando em conta as sugestões dos comentários.
4. **“Existe evidência suficiente para essa afirmação”**: Afirmações com mais de 75% de concordância foram consideradas suficientemente embasadas para serem publicadas.

Qualquer afirmação que não atendesse a esses quatro critérios era levada para a terceira fase para revalidação. Portanto, para ser considerada parte do Consenso e retirada da próxima fase, era necessário que uma afirmação passasse por todos esses critérios.

Nesta fase, o número de afirmações que necessitavam de revalidação diminuiu para 42, refletindo o aprimoramento significativo do conteúdo e da clareza das afirmações sobre o lipedema.

### Análise estatística do estudo de Consenso de Lipedema

**Fase 1:** A primeira Fase do nosso estudo foi de natureza descritiva, sem a realização de análise estatística. O foco estava na geração e discussão das afirmações sobre o lipedema, baseando-se na revisão da literatura e na experiência dos especialistas envolvidos.
**Fases 2 e 3:** Durante as Fases 2 e 3 do estudo, implementamos análises estatísticas específicas para avaliar as afirmações sobre o lipedema. Utilizamos uma escala Likert de 5 pontos, em que 1 significava “Discordo completamente” e 5, “Concordo totalmente”. Para cada afirmação, somamos os pontos atribuídos por todos os participantes, formando uma pontuação total para cada item.

A partir dessas pontuações totais, calculamos uma média ponderada que considerou o número de respostas para cada afirmação. Essa média ponderada foi calculada para as métricas definidas: “Concordância com a afirmação”, “A afirmação deve ser incluída no texto final”, “A afirmação está clara” e “Existe evidência suficiente para essa afirmação”.

Estabelecemos um limiar de 75% de concordância, baseado na média ponderada, para que uma afirmação fosse considerada parte do consenso. As afirmações que atingiram esse patamar em qualquer das métricas foram aceitas para inclusão no consenso ou para publicação. Aquelas que não alcançaram essa porcentagem foram revisadas e submetidas a uma nova avaliação na fase seguinte.

## RESULTADOS

### Fase 1

Na primeira fase do nosso estudo de consenso sobre lipedema, elaboramos um total de 90 afirmações. Essas afirmações foram cuidadosamente redigidas com base na *expertise* acumulada dos especialistas envolvidos e nas publicações atuais sobre o tema. O conjunto de afirmações abrangeu uma ampla gama de tópicos relacionados ao lipedema, incluindo aspectos de diagnóstico, tratamento, manejo clínico e compreensão da fisiopatologia da condição.

### Fase 2

Na segunda fase do estudo de consenso sobre lipedema, tivemos a participação espontânea de 113 especialistas de diversas áreas da saúde, distribuídos geograficamente por todo o Brasil. Os participantes tinham, em média, 21,6 anos de experiência na medicina e 8 anos de atuação no tratamento de pacientes com lipedema. A especialidade predominante foi Cirurgia Vascular, representando 83,2% dos respondentes, seguida por Angiologia (8%), Cirurgia Plástica (1,8%), Fisioterapia (2,7%), Educação Física (1,8%), Nutrição (1,8%), Endocrinologia (0,9%) e Ginecologia (0,9%) (Figura 2).

**Figura 2:** Distribuição de especialidades médicas e profissionais de saúde.

A distribuição dos participantes por estado brasileiro mostrou uma maior concentração em São Paulo, com 31,86% dos respondentes, além de representatividade em várias outras regiões do país, como Minas Gerais e Rio de Janeiro, com 9,73% cada, e Bahia, com 5,31%, entre outros (Figura 3).

**Figura 3:** Distribuição demográfica dos participantes do consenso.

Quanto à atuação na gestão do lipedema, a maioria dos participantes (91,15%) atua no tratamento clínico da condição, com outras abordagens terapêuticas como alimentação (45,13%), atividade física (47,79%) e fisioterapia (32,74%) também sendo comumente empregadas. Um percentual menor de participantes relatou atuação em Cirurgia (15,93%) e Diagnóstico por imagem (35,40%).

Ao final desta fase, 33 afirmações não alcançaram consenso ou apresentaram problemas de clareza, sendo selecionadas para revisão e reavaliação na fase seguinte do estudo. Foram criadas mais nove afirmações baseadas em sugestões dos participantes. Esse processo de refinamento é essencial para assegurar que as afirmações finais sejam claras, precisas e reflitam o consenso da comunidade médica sobre o manejo do lipedema.

Os resultados que alcançaram consenso na segunda fase do estudo estão apresentados na Tabela 1^9-79^ . As afirmações que não atingiram consenso foram revisadas, reescritas e posteriormente submetidas a avaliação na terceira fase.

**Tabela 1:** Resultados da segunda fase.

Afirmação	Tema	Concordância	Inclusão	Clareza	Evidência	Respondidos/pulados	Nível de evidência
O lipedema é uma doença crônica e sistêmica^9,10^	Etiologia	4,31	4,19	4,2	3,96	110/3	B
A prevalência do lipedema no Brasil é de 12,3% da população feminina adulta^4^	Epidemiologia	3,78	3,81	3,98	3,49	109/4	B
O lipedema é uma doença complexa com necessidade de tratamento multiprofissional^11,12^	Abordagem multidisciplinar	4,66	4,62	4,6	4,45	106/7	B
O lipedema normalmente se apresenta como um aumento simétrico e bilateral do tecido adiposo subcutâneo nas extremidades^13,14^	Características clínicas	4,2	4,17	4,13	4,04	106/7	B
O lipedema se caracteriza por uma expansão desproporcional do tecido adiposo subcutâneo das extremidades em relação ao tronco^12,15^	Patogênese/fisiopatologia	4,29	4,15	4,18	4,12	106/7	B
O lipedema pode envolver um excesso de depósito de tecido adiposo nas extremidades superiores, simetricamente e bilateralmente^10,12,13^	Patogênese/fisiopatologia	3,98	3,92	4,02	3,85	105/8	B
O lipedema normalmente poupa mãos e pés^14,16^	Características clínicas	4,32	4,26	4,34	4,19	104/9	B
A sensibilidade dolorosa à pressão e/ou ao alongamento é observada, por exemplo, pela palpação e é relatada principalmente pelos pacientes como dor^17,18^	Características clínicas	4,39	4,34	4,37	4,2	102/11	B
Pacientes frequentemente relatam inchaço ou sensação de peso nas áreas afetadas^4,12,19,20^	Características clínicas	4,4	4,35	4,31	4,21	102/11	B
O edema de depressão (sinal de Godet ou cacifo) geralmente não está presente no tecido afetado pelo lipedema^12,13,21^	Diagnóstico	4,12	4,08	4,14	4,05	100/13	B
A elevação da extremidade afetada pelo lipedema geralmente não resulta em uma redução do volume^22,23^	Diagnóstico	4,15	4,13	4,19	4,12	99/14	B
Pacientes com lipedema geralmente experimentam fácil formação de hematomas^4,12,23,24^	Características clínicas	4,07	4,09	4,14	3,97	99/14	B
O sinal de Kaposi-Stemmer geralmente é negativo no lipedema^25,26^	Diagnóstico	4,27	4,24	4,25	4,15	97/16	B
O lipedema é uma doença que envolve tecido adiposo subcutâneo^15,27,28^	Características clínicas	4,29	4,21	4,22	4,13	97/16	B
Vários achados sugerem que a inflamação pode contribuir para a patogênese do lipedema^20,21,29^	Patogênese/fisiopatologia	4,25	4,2	4,22	4,08	96/17	B
Vários achados sugerem que os fatores hormonais podem contribuir para a patogênese do lipedema^9,12,30,31^	Patogênese/fisiopatologia	4,37	4,37	4,32	4,13	95/18	B
Vários achados sugerem que o conteúdo de fluido extracelular pode estar elevado no tecido afetado pelo lipedema em comparação com controles saudáveis^32-34^	Patogênese/fisiopatologia	4,1	3,98	3,98	3,89	93/20	C
O lipedema afeta principalmente as mulheres biológicas. A ocorrência em homens parece ser possível, mas é rara^25,35,36^	Características clínicas	4,23	4,15	4,2	4,09	92/21	B
Mudanças hormonais, como durante a puberdade, gravidez e menopausa, podem desencadear ou exacerbar os sintomas do lipedema^10,12,25^	Características clínicas	4,46	4,41	4,46	4,34	93/20	B
Vários achados sugerem que o lipedema é hereditário^25,37-39^	Etiologia	4,26	4,26	4,28	4,13	93/20	B
A obesidade é uma doença coincidente frequentemente observada em pacientes com lipedema^4,12,23^	Epidemiologia	3,99	3,9	4,04	3,9	93/20	B
O lipedema e a obesidade não são mutuamente causais^12,40^	Patogênese/fisiopatologia	3,86	3,79	3,86	3,66	93/20	B
O índice de massa corporal (IMC) tem um valor limitado para distinguir entre lipedema e obesidade	Diagnóstico	4,13	4,1	4,16	3,98	93/20	B
Em casos em que o lipedema coincide com a obesidade, pode-se esperar que os sintomas do lipedema persistam após cirurgia bariátrica^25,40,41^	Prognóstico	4,22	4,15	4,17	4,01	93/20	C
O lipedema pode impactar negativamente a saúde mental e a qualidade de vida em geral^4,42^	Aspectos sociais e psicológicos	4,66	4,59	4,62	4,47	87/26	B
Um diagnóstico atrasado ou o tratamento tardio do lipedema afeta negativamente a carga de sintomas do paciente, o bem-estar mental e a qualidade de vida em geral^43-45^	Prognóstico	4,45	4,38	4,38	4,26	87/26	C
O diagnóstico clínico de lipedema depende do histórico médico do paciente, exame físico e exclusão de diagnóstico diferencial^23,27,46^	Diagnóstico	4,44	4,41	4,44	4,32	87/26	B
Todas as intervenções terapêuticas visam aliviar os sintomas e prevenir ou retardar a progressão da doença^11,47^	Tratamento	4,14	4,15	4,2	4,04	81/32	B
O cuidado multidisciplinar envolvendo médicos, fisioterapeutas, nutricionistas e profissionais de saúde mental é essencial para o gerenciamento abrangente. As especialidades médicas que devem atuar no lipedema são: Cirurgia Vascular, Endocrinologia, Ortopedia, Cirurgia Plástica, Psiquiatria, Ginecologia, entre outras^48-50^	Abordagem multidisciplinar	4,48	4,46	4,46	4,39	79/34	B
A dor do lipedema e a sensibilidade física nas áreas afetadas pelo lipedema foram relatadas como sendo reduzidas pela bandagem, compressão, terapia física descongestionante complexa (CTD), mudanças dietéticas, exercícios de baixo impacto e lipoaspiração, com tamanhos e durações de efeito variáveis^51-55^	Tratamento	4,38	4,3	4,34	4,14	79/34	C
A gestão conservadora do lipedema deve incluir mudanças de estilo de vida e nutrição, terapia de compressão e exercício de baixo impacto para aliviar os sintomas e melhorar a qualidade de vida^23,38,54,55^	Tratamento	4,52	4,47	4,48	4,33	79/34	C
A CDT, com pelo menos alguns de seus elementos, pode ser um tratamento importante e eficaz até mesmo para lipedema em estágio inicial^56-58^	Tratamento	4,26	4,18	4,25	4,08	78/35	C
A orientação nutricional pode ajudar os pacientes a gerenciar seu peso, otimizar a saúde geral, reduzir os sintomas associados ao lipedema e melhorar sua resposta às intervenções terapêuticas^12,40^	Tratamento	4,55	4,5	4,53	4,42	78/35	C
Considerando que a obesidade grave piora as manifestações do lipedema, a gestão da doença deve incluir a normalização do peso, com foco na proporção cintura-altura (WHtR)^59,60^	Tratamento	3,98	3,88	4	3,81	77/36	C
Apoio psicológico e social, abordando questões de imagem corporal, bem-estar mental e estratégias de enfrentamento, podem ser importantes para lidar com a carga de sintomas dos pacientes que vivem com lipedema^42,61-64^	Aspectos sociais e psicológicos	4,59	4,54	4,6	4,45	76/37	C
Exercícios de baixo impacto, como atividade física na água, caminhada e ioga, podem ajudar a manter a mobilidade e apoiar o gerenciamento de peso em indivíduos com lipedema^54,55^	Tratamento	4,53	4,49	4,5	4,37	76/37	C
Nos casos em que o lipedema coincide com obesidade e doença metabólica, é aconselhável priorizar a cirurgia bariátrica antes de considerar a lipoaspiração^10,21,65-68^	Prevenção/prognóstico	3,99	3,99	4,05	3,84	75/38	C
A lipoaspiração com preservação linfática deve ser considerada quando há potencial para impacto significativo no bem-estar físico^21,67,68^	Tratamento	4,12	4,11	4,15	4	76/37	C
As intervenções cirúrgicas devem ser realizadas por centros experientes com experiência comprovada na gestão do lipedema, incluindo tratamentos conservadores, como parte de uma abordagem integral^10,12,67^	Tratamento	4,42	4,41	4,45	4,27	76/37	C
Aumentar a conscientização sobre o lipedema dentro da comunidade médica e da sociedade é essencial para reduzir o diagnóstico errado e o estigma^12,14^	Abordagem multidisciplinar	4,62	4,64	4,67	4,5	76/37	C
São necessárias mais pesquisas para elucidar os mecanismos biológicos subjacentes do lipedema, levando a critérios de diagnóstico objetivos e terapias direcionadas^69^	Pesquisa e desenvolvimento	4,66	4,65	4,63	4,55	76/37	C
Estudos de longo prazo são necessários para avaliar a eficácia e a segurança das modalidades de tratamento para lipedema. Em particular, o tratamento cirúrgico deve ser estudado para seu impacto metabólico a curto, médio e longo prazo. O impacto psicológico também deve ser estudado^12,55,69^	Pesquisa e desenvolvimento	4,68	4,64	4,61	4,6	76/37	C
Esforços colaborativos entre pacientes, pesquisadores, clínicos e grupos de apoio são fundamentais para avançar o conhecimento	Pesquisa e desenvolvimento	4,68	4,69	4,72	4,65	76/37	C
A dieta anti-inflamatória e a dieta cetogênica têm demonstrado melhora sintomática em determinadas pacientes^19,51^	Tratamento	4,16	4,13	4,19	3,89	76/37	C
Os sintomas do lipedema podem ser agravados por longos períodos de inatividade ou mesmo viagens aéreas^23,62^	Características clínicas	4,04	4,08	4,03	3,75	76/37	C
O aumento do tecido adiposo nos braços e pernas em pacientes com lipedema pode dificultar as atividades diárias^12,54,55^	Características clínicas	4,55	4,53	4,5	4,22	75/38	C
Os pacientes com lipedema muitas vezes relatam uma sensibilidade exagerada nas áreas afetadas^10,12,41^	Características clínicas	4,45	4,41	4,47	4,26	73/40	B
Os pacientes com lipedema podem apresentar dificuldades para encontrar roupas adequadas devido ao aumento das extremidades^70-73^	Aspectos sociais e psicológicos	4,58	4,4	4,54	4,22	73/40	C
A dor relacionada ao lipedema pode interferir no sono e no descanso adequado^4,74-77^	Características clínicas	4,19	4,13	4,24	3,9	72/41	C
A identificação precoce e o tratamento adequado do lipedema podem ajudar a minimizar a progressão da doença^12,13^	Prevenção/prognóstico	4,45	4,44	4,42	4,29	73/40	C
A autoconsciência e a educação em relação ao lipedema são essenciais para o cuidado e manejo proativos em casa^10,61,78^	Aspectos sociais e psicológicos	4,58	4,49	4,48	4,36	73/40	C
A terapia de massagem manual pode ajudar a aliviar os sintomas de inchaço e dor associados ao lipedema	Tratamento	4,26	4,21	4,21	3,99	73/40	C
Pessoas com lipedema têm maiores riscos de desenvolverem transtornos internos dos joelhos como condromalácia e artrose precoce^4,9,25^	Características clínicas	4,11	4,06	4,09	3,91	71/42	C
No programa de exercícios físicos, um professor de educação física com conhecimento acerca do lipedema é essencial para a prevenção de lesões^54,55^	Abordagem multidisciplinar	4,44	4,31	4,39	4,1	68/45	C
A decisão pela indicação cirúrgica deve ser aprovada pelo médico clínico, pelo médico cirurgião e pela paciente. Deve ter o objetivo de melhora da mobilidade em primeiro lugar e melhora sintomática em segundo lugar. O resultado estético não deve ser o objetivo principal do tratamento cirúrgico do lipedema^10^	Tratamento	4,09	4,13	4,23	3,94	65/48	C
Em casos em que o lipedema coincide com a obesidade, pode-se esperar que os sintomas do lipedema persistam após cirurgia bariátrica^12,40,66^	Prognóstico	4,38	4,35	4,38	4,1	61/52	

Nível de evidência A: Ensaios clínicos randomizados/metanálises; B: Casos-controle/um ensaio clínico/coorte; C: Relatos de casos/consenso de especialistas.

### Fase 3

Na fase final do nosso estudo de consenso, contamos com a participação de 59 especialistas. Durante essa etapa, 42 afirmações foram avaliadas com o objetivo de estabelecer um consenso robusto sobre o lipedema.

Das 42 afirmações consideradas (Tabela 2)^4,5,9,10,12,13,20,21,26,27,29,30,32,34,37-39,42,47,49,53,54,60-64,74,76-78,80-108^ , todas alcançaram consenso significativo nos critérios de “Concordância com a afirmação”, “A afirmação deve ser incluída no texto final” e “A afirmação está clara”, indicando uma forte concordância geral entre os especialistas sobre o conteúdo e a relevância das afirmações para o consenso final.

**Tabela 2:** Resultados da terceira fase.

Afirmação	Tema	Concordância	Inclusão	Clareza	Evidência	Respondidos/pulados	Nível de Evidência
A prevalência do lipedema avançado não é bem conhecida devido à falta de estudos abrangentes e ao subdiagnóstico. Muitos casos podem ser confundidos com obesidade, linfedema ou doença venosa crônica. É crucial identificar a condição precocemente para prevenir a progressão sem tratamento adequado. Mais pesquisas são necessárias para entender sua verdadeira extensão e melhorar o acompanhamento dessas pacientes^63^	Epidemiologia	4,75	4,6	4,6	4,24	55/3	C
A dor e sensibilidade aumentadas no lipedema costumam se concentrar nas áreas com excesso de tecido adiposo, mas também podem afetar outras áreas próximas a nervos sensíveis ou devido à inflamação sistêmica. Isso pode levar a diagnósticos equivocados, como fibromialgia. Portanto, uma avaliação cuidadosa é crucial para entender corretamente a distribuição e a causa da dor nesses pacientes^20,29,80^	Características clínicas	4,52	4,44	4,4	4	55/3	C
O lipolinfedema é uma condição que ocorre quando o linfedema surge como uma complicação do lipedema devido à inflamação crônica. No entanto, se o linfedema tem outra origem, como sedentarismo ou sobrepeso, ele é considerado uma comorbidade, e não um estágio do lipolinfedema^29,47,81-83^	Características clínicas	3,95	3,87	4,15	3,69	55/3	B
Alterações no sistema de pequenos vasos sanguíneos ou disfunção microvascular parecem desempenhar um papel na origem do lipedema, podendo estar presentes em todas as fases da condição^12,30,84,85^	Patogênese/fisiopatologia	3,85	3,87	3,96	3,57*	52/6	C
A sensibilidade e a dor podem ser exacerbadas por fatores como o excesso de esforço nas articulações, que pode resultar de um peso corporal elevado, ou por irritação da pele causada por atrito ou inflamação, podendo ser ainda mais intensificadas em presença de comorbidades como a fibromialgia^20,29,62,80^	Características clínicas	4,37	4,33	4,31	3,94	52/6	C
O lipedema não é causado pela obesidade nem a obesidade é causada pelo lipedema; são condições distintas com seus próprios processos fisiopatológicos. Contudo, a obesidade pode agravar os sintomas do lipedema, e um aumento de peso significativo pode atuar como um gatilho para o aparecimento do lipedema. É importante que os indivíduos, independentemente do seu peso, busquem avaliação médica se suspeitam da condição, já que o tratamento precoce pode ser benéfico. Mais estudos são necessários para entender completamente a relação entre as duas condições^12^	Patogênese/fisiopatologia	4,46	4,4	4,48	4	52/6	C
A linfoestase pode ocorrer em qualquer fase do lipedema, mas é mais evidente e frequente nos estágios mais avançados da doença^34,39^	Patogênese/fisiopatologia	4,36	4,36	4,43	4,12	50/8	C
Pessoas com lipedema, especialmente aquelas que mantêm um peso corporal dentro dos parâmetros saudáveis, podem apresentar um perfil metabólico comparável ao da população em geral, incluindo um menor risco para condições como hipercolesterolemia e potencialmente menor risco para doenças cardiovasculares^4,9,86,87^	Epidemiologia	4,02	4,02	4,1	3,76	50/8	C
Estudos sugerem que a incidência de hipotireoidismo pode ser superior em pacientes com lipedema comparativamente a grupos sem a condição^4^	Pesquisa e desenvolvimento	3,76	3,74*	3,98	3,6*	50/8	C
O lipedema pode estar relacionado a condições do tecido conjuntivo, como a síndrome de Ehlers-Danlos, especialmente no que tange a variações na gravidade da hipermobilidade articular^26,37,88^	Patogênese	3,81	3,8	3,92	3,57*	49/9	C
O lipedema pode estar associado à fraqueza muscular, afetando tanto a capacidade de ganhar massa muscular quanto a função muscular em si, tanto nas partes proximais quanto distais dos membros superiores e inferiores^54,89,90^	Características clínicas	4	4,06	4,16	3,84	49/9	C
O impacto psicológico em pacientes com lipedema parece ser mais frequentemente uma consequência dos sintomas da condição do que uma causa inicial deles. No entanto, é importante considerar a saúde mental como parte integrante do manejo do lipedema, já que pode existir uma interação contínua entre o estado psicológico e os sintomas físicos da doença^4,42,61,64^	Aspectos sociais e psicológicos	4,65	4,59	4,59	4,35	49/9	C
Durante exames clínicos de rotina, é recomendável a utilização de medidas antropométricas mais específicas, como a proporção cintura-altura e a relação cintura-quadril, para uma avaliação mais precisa. Métodos adicionais, como densitometria corporal total ou bioimpedância, podem ser considerados para fornecer uma compreensão mais detalhada da composição corporal, em vez de depender apenas do índice de massa corporal, que pode não refletir adequadamente as características particulares do lipedema^60,91^	Diagnóstico	4,63	4,53	4,61	4,35	49/9	C
A classificação do lipedema em estágios é baseada na distribuição e progressão do tecido adiposo, e não necessariamente indica a intensidade dos sintomas, que podem variar de acordo com a inflamação e comorbidades associadas. Pacientes em estágios avançados podem relatar mais sintomas, mas a experiência de dor e outras queixas é individual e pode não estar diretamente relacionada ao estágio visual da doença^10,12,92^	Classificação	4,64	4,64	4,62	4,27	45/13	C
A classificação clínica do lipedema em estágios pode influenciar a escolha das técnicas de tratamento, com intervenções cirúrgicas variando de acordo com o estágio da condição. Enquanto os estágios iniciais podem não exigir cirurgia, os estágios mais avançados, especialmente quando há restrição significativa à mobilidade, podem requerer abordagens cirúrgicas específicas^10,12,49,78,92^	Classificação	4,4	4,42	4,56	4,56	45/13	C
A progressão dos sintomas do lipedema pode ser atenuada ou mesmo evitada com o tratamento adequado e hábitos de vida saudáveis	Tratamento	4,71	4,69	4,69	4,47	45/13	C
No lipedema, o aumento do volume dos membros é característico e pode contribuir para um maior peso corporal, diferenciando-se da obesidade comum pela sua distribuição e resistência à perda de peso convencional^9,12,93^	Características clínicas	4,51	4,5	4,58	4,31	45/13	C
As classificações clínicas baseadas na localização do lipedema são úteis para descrever a distribuição da doença e podem orientar o diagnóstico e o planejamento do tratamento, sugerindo também possíveis evoluções e complicações relacionadas^10,12,77^	Classificação	4,29	4,24	4,24	3,93	45/13	C
Práticas de gerenciamento do estresse, como *mindfulness* e técnicas de respiração, contribuem para o bem-estar geral e podem melhorar a adesão ao tratamento do lipedema, além de ajudar a manejar o impacto emocional e a dor associados à condição^61,64^	Tratamento	4,31	4,18	4,33	3,98	45/13	C
É comum observar joelhos em valgo em indivíduos com lipedema, o que pode afetar a marcha e aumentar o risco de complicações como gonartrose, impactando diretamente a qualidade de vida e a mobilidade^9,76^	Características clínicas	4,36	4,36	4,41	4	45/13	C
Frequentemente, indivíduos com lipedema apresentam um consumo elevado de carboidratos e alimentos potencialmente inflamatórios, o que pode levar a uma ingestão insuficiente de proteínas conforme as recomendações diárias^94-96^	Abordagem multidisciplinar	4,36	4,33	4,49	4,09	45/13	C
Indivíduos com lipedema sintomático frequentemente apresentam sensibilidades alimentares^38^	Características clínicas	3,91	3,86	4,09	3,7*	45/13	C
Indivíduos com lipedema sintomático tendem a levar um estilo de vida sedentário, embora o exercício, quando praticado, deva ser bem escolhido para evitar atividades de alto impacto que podem piorar os sintomas^97,98^	Características clínicas	4,44	4,33	4,4	4,09	45/13	C
É observada uma incidência de alergias respiratórias, como rinite e sinusite, em pessoas com lipedema sintomático^99^	Características clínicas	3,6	3,58*	3,88*	3,49*	45/13	C
Pessoas com lipedema podem apresentar mudanças nos hábitos intestinais, muitas vezes como resultado de uma dieta com alimentos inflamatórios e alterações na flora intestinal, que podem contribuir para um estado inflamatório sistêmico^95,100^	Características clínicas	4,05	4,05	4,23	3,84	45/13	C
Pessoas com lipedema, especialmente em estágios mais avançados, enfrentam desafios no uso de meia elástica e métodos de elastocompressão devido à desproporção significativa dos membros e ao desconforto causado pela compressão, que pode ser intensificado em períodos de maior inflamação. É importante que tais dispositivos sejam adaptados individualmente^30,82,101^	Tratamento	4,65	4,58	4,6	4,28	45/13	C
Indivíduos com lipedema sintomático frequentemente relatam distúrbios do sono^4,74^	Características clínicas	3,81	3,67	4,05	3,56	45/13	C
Indivíduos com lipedema sintomático muitas vezes não atendem à recomendação diária de ingestão de água	Características clínicas	4,02	3,98	4,23	3,79	43/15	C
Pessoas com lipedema sintomático muitas vezes consomem fibras abaixo da quantidade diária recomendada	Características clínicas	4,17	4,05	4,31	3,88	42/16	C
Embora a conexão entre lipedema e transtorno de déficit de atenção e hiperatividade tenha sido proposta, ela ainda necessita de mais pesquisas. Frequentemente, pessoas com lipedema apresentam impulsividade e requerem suporte psicológico. A cirurgia é indicada quando esse traço impulsivo está sob controle. Adicionalmente, o lipedema pode estar associado a outros transtornos de saúde mental^61^	Aspectos psicológicos e sociais	3,93	3,9	4,1	3,64	42/16	C
Pacientes jovens em estágios iniciais apresentam bons resultados pós-cirurgia, mas o tratamento conservador é vantajoso para todos. Sempre é necessário considerar a indicação cirúrgica e os efeitos funcionais, além dos estéticos. Impactos na fertilidade ainda são incertos^5,53,77^	Tratamento	3,95	3,95	3,9	3,67*	42/16	C
A decisão para um procedimento cirúrgico no tratamento do lipedema não deve ser tomada precipitadamente. Ela não deve ser considerada antes de 1 ano de tratamento clínico, sempre priorizando a compreensão da paciente sobre sua condição. No entanto, dependendo do grau da doença e da resposta do paciente ao tratamento clínico, a cirurgia pode ser benéfica em estágios avançados para melhorar a mobilidade do paciente^5,10,102^	Tratamento	4,46	4,45	4,49	4,27	41/17	C
A progressão do lipedema se dá durante episódios de inflamação, nos quais a doença avança. Sem inflamação, a condição permanece estável^21,32^	Tratamento	4,08	3,9	4,15	3,75	40/18	C
O diagnóstico do lipedema geralmente envolve uma avaliação inicial que inclui ultrassom para descartar edema, eco-Doppler para descartar varizes e fotopletismografia para avaliar a função venosa. Se esses exames não indicarem problemas vasculares, é possível considerar a alta do ponto de vista venoso^27,103,104^	Diagnóstico	3,63	3,63	3,95	3,39*	38/20	C
A deficiência de vitamina D é uma ocorrência frequente em mulheres com lipedema, destacando a importância da monitorização e suplementação adequada dessa vitamina para a saúde dessas pacientes^105,106^	Tratamento	4,13	4,15	4,28	3,92	39/19	C
Atividades de fortalecimento muscular, especialmente direcionadas à musculatura posterior da coxa, têm demonstrado ser benéficas para mulheres com lipedema, contribuindo para melhorar a qualidade de vida e o manejo dos sintomas dessa condição^54,90^	Tratamento	4,29	4,28	4,41	4,05	39/19	C
A taxa de metabolismo basal em pacientes com lipedema sintomático tende a ser inferior ao esperado, o que pode influenciar nas estratégias de gerenciamento e no acompanhamento nutricional dessas pacientes^86^	Multidisciplinar	4,03	3,97	4,18	3,79	39/19	C
O autocuidado no tratamento do lipedema pode incluir o uso de escovas de cerdas macias para automassagem e a utilização de plataformas vibratórias e meias elásticas^10,107,108^	Tratamento	4,23	4,15	4,26	3,95	39/19	C
É recomendado o uso rotineiro de questionários de qualidade de vida, como o Questionário de Avaliação de Sintomas de Lipedema (QuASiL), para avaliar a eficácia das intervenções terapêuticas no tratamento do lipedema^92^	Tratamento	4,56	4,51	4,59	4,31	39/19	C
Avaliar gatilhos de agudização do lipedema, como questões alimentares e hormonais e estressores sociais e profissionais, pode contribuir para um melhor controle da doença^21^	Diagnóstico	4,67	4,59	4,67	4,44	39/19	C
Existe uma relação observada entre o lipedema e a presença de varizes; entretanto, essa associação necessita de investigações mais aprofundadas. O tratamento da doença venosa associada deve considerar a presença do lipedema e estabelecer uma relação causal dos sintomas^4,12,13,101^	Pesquisa e desenvolvimento	4,23	4,18	4,26	4,05	39/19	C
Não é adequado induzir os pacientes com lipedema a realizar numerosos tratamentos custosos sem evidências científicas claras que sustentem sua eficácia	Tratamento	4,87	4,82	4,82	4,62	39/19	C

Nível de evidência A: Ensaios clínicos randomizados/metanálises; B: Casos-controle/um ensaio clínico/coorte; C: Relatos de casos/consenso de especialistas.

No entanto, nove dessas afirmações não atingiram o nível de evidência considerado suficiente para a métrica “Existe evidência suficiente para essa afirmação”. Isso sugere que, apesar da concordância sobre a validade dessas afirmações entre os especialistas, existe uma necessidade reconhecida de mais pesquisas ou dados para apoiar plenamente essas particularidades sobre o lipedema.

Não houve sugestões de inclusão de novas afirmações, o que indica que o conjunto de afirmações propostas foi considerado abrangente e satisfatório pelos participantes para representar o conhecimento atual sobre o lipedema.

Esse resultado demonstra um passo significativo na consolidação do entendimento sobre o lipedema, estabelecendo uma base sólida para a prática clínica e diretrizes futuras. No entanto, também destaca áreas que necessitam de investigação adicional para fortalecer a base de evidências sobre a condição.

Os resultados da segunda fase estão apresentados na Tabela 2, e os temas abordados são mostrados na Figura 4.

**Figura 4:** Temas abordados pelas afirmativas.

## DISCUSSÃO

Este consenso brasileiro sobre lipedema representa um esforço colaborativo significativo para delinear as características, o diagnóstico e o tratamento dessa condição complexa. A natureza multifacetada do lipedema, que envolve diversos mecanismos corporais e apresenta manifestações variadas entre os pacientes, torna a obtenção de um consenso um desafio notável. O lipedema, sendo uma doença crônica sem cura conhecida, exige uma abordagem multidisciplinar para seu manejo, envolvendo especialistas de várias áreas da saúde.

As afirmações consensuais deste trabalho ressaltam que o lipedema está longe de ser uma condição simples ou unidimensional. A doença interage com vários sistemas corporais, influenciando a saúde mental, a funcionalidade física e a qualidade de vida dos pacientes. O lipedema não deve ser reduzido a uma “doença da gordura” nem confundido com outras comorbidades, pois possui características únicas e distintas. É evidente a necessidade de mais pesquisas para compreender completamente a patologia do lipedema, especialmente em relação aos mecanismos fisiopatológicos e às melhores práticas de tratamento.

### Epidemiologia e características clínicas

A prevalência do lipedema no Brasil, estimada em 12,3%^4^ da população feminina adulta, ressalta a relevância dessa condição para a saúde pública e a necessidade de maior reconhecimento médico. Essa taxa indica que o lipedema é uma condição bastante comum, mas muitas vezes é subdiagnosticado ou confundido com outras condições, como a obesidade. A sobreposição com a obesidade é frequente e pode complicar tanto o diagnóstico quanto o tratamento do lipedema, pois ambas as condições podem coexistir e interagir de maneiras que afetam a saúde geral do paciente^4,12,23^ .

A falta de clareza em relação à prevalência do lipedema avançado destaca uma lacuna significativa no conhecimento médico atual e aponta para a necessidade de mais pesquisas epidemiológicas. A distinção entre lipedema e condições similares, como o linfedema e doenças venosas crônicas, é complicada pela sobreposição dos sintomas e pela falta de conscientização sobre o lipedema. Isso enfatiza a importância de uma abordagem diagnóstica mais refinada e da implementação de protocolos de avaliação que possam diferenciar efetivamente entre essas condições^63^ .

A detecção precoce do lipedema é essencial para prevenir a progressão para estágios mais avançados, que são mais difíceis de gerir e têm um impacto significativo na qualidade de vida e na mobilidade. A identificação e o tratamento precoces podem ajudar a mitigar o desenvolvimento de complicações e a deterioração funcional^12^ .

Curiosamente, a observação de que algumas pessoas com lipedema que mantêm um peso saudável possuem um perfil metabólico comparável ao da população geral é um ponto crucial que desafia muitos preconceitos associados ao lipedema. Isso pode indicar que o lipedema, por si só, sem outras condições coexistentes, como a obesidade, pode não estar associado a um risco aumentado de comorbidades metabólicas^4,9,86,87^ . No entanto, é importante ressaltar que a presença de lipedema pode influenciar a capacidade de exercício e o estilo de vida, que são fatores-chave na manutenção de um perfil metabólico saudável^97,98^ .

Os especialistas consultados foram consensuais de que o lipedema possui características clínicas particulares que precisam ser reconhecidas. Essa condição é caracterizada por um aumento simétrico e bilateral do tecido adiposo subcutâneo nas pernas e braços, normalmente poupando as mãos e os pés^10^ . Esse acúmulo de gordura não se limita a uma questão estética; trata-se de uma condição dolorosa e sensível ao toque, que pode ser severamente debilitante^14,16^ . Os pacientes frequentemente relatam dor e uma sensação de peso nas áreas afetadas, além de uma propensão a desenvolver hematomas com facilidade, o que pode indicar uma vulnerabilidade do tecido conectivo ou vascular^17,18^ .

Embora o lipedema seja uma doença predominantemente feminina, com um nexo significativo com as flutuações hormonais experimentadas durante eventos como a puberdade, gravidez e menopausa, casos raros em homens foram observados. Esses eventos hormonais podem atuar como catalisadores para o início ou agravamento dos sintomas do lipedema^9,12,30,31^ .

A condição pode ser exacerbada por períodos de inatividade ou viagens aéreas prolongadas, sugerindo que o movimento regular é benéfico para a gestão dos sintomas. Além disso, o aumento do tecido adiposo pode dificultar atividades do dia a dia e até mesmo a escolha de roupas adequadas, contribuindo para o estresse psicológico e problemas de autoimagem^70-73^ .

Os pacientes também podem relatar uma sensibilidade exagerada nas áreas afetadas, com a dor do lipedema interferindo significativamente na capacidade de realizar atividades cotidianas e na qualidade do sono^20,29,80^ . Dessa forma, o lipedema vai além de uma condição física, influenciando amplamente a saúde mental e a qualidade de vida dos pacientes. A autoconsciência e a educação sobre a doença são essenciais para que os pacientes possam adotar estratégias de autocuidado e manejo proativos, visando uma melhor qualidade de vida^10,61,78^ .

A clínica do lipedema é complexa e multifacetada, com várias manifestações que podem afetar significativamente o bem-estar dos pacientes. A dor e a sensibilidade aumentadas são características centrais do lipedema e frequentemente se localizam nas áreas com excesso de tecido adiposo^20,29,80^ . Esses sintomas podem ser confundidos com outras condições, como a fibromialgia, devido à semelhança na distribuição da dor. No entanto, é importante diferenciar essas duas condições para garantir uma gestão eficaz^20,29,62,80^ .

O lipolinfedema representa a progressão no espectro do lipedema, em que o linfedema se desenvolve como uma consequência da condição inflamatória crônica subjacente. Diferenciar a origem do linfedema é crucial, pois o tratamento e as implicações a longo prazo podem variar significativamente^29,47,81-83^ . A dor no lipedema pode ser exacerbada pelo excesso de carga nas articulações, que pode resultar do peso corporal elevado, e pela irritação da pele devido ao atrito ou inflamação^9,76^ . Esses fatores são agravados pela presença de comorbidades, como fibromialgia, que podem intensificar a experiência da dor^20,29,80^ .

Os pacientes com lipedema podem apresentar fraqueza muscular, que afeta tanto a capacidade de ganhar massa muscular quanto a função muscular. Isso pode comprometer a mobilidade e a capacidade de realizar atividades diárias, aumentando o risco de sedentarismo^54,89,90^ . A distribuição atípica do tecido adiposo no lipedema é distinta da obesidade convencional e muitas vezes é resistente a métodos tradicionais de perda de peso, como dieta e exercício. Isso pode levar a um ciclo de ganho de peso e frustração para o paciente^9,12,93^ .

Mudanças ortopédicas, como joelhos em valgo, são comuns em pacientes com lipedema e podem afetar a marcha e aumentar o risco de complicações ortopédicas, como a gonartrose, afetando a qualidade de vida e a mobilidade^9,76^ . Além disso, sensibilidades alimentares e alterações nos hábitos intestinais podem indicar uma resposta inflamatória sistêmica que exacerba os sintomas do lipedema. A ocorrência de alergias respiratórias e distúrbios do sono também é relatada com frequência e pode estar relacionada ao estado inflamatório crônico. A ingestão inadequada de água e fibras pode contribuir para as dificuldades intestinais e para o manejo geral da doença. Portanto, é essencial que os pacientes com lipedema mantenham uma dieta balanceada e atendam às recomendações diárias de ingestão de nutrientes para ajudar a mitigar esses efeitos.

O reconhecimento e a compreensão desses aspectos clínicos são fundamentais para que os profissionais de saúde possam fornecer um suporte abrangente e individualizado aos pacientes com lipedema. O objetivo deve ser não apenas o manejo dos sintomas, mas também a melhoria geral da qualidade de vida.

### Aspectos sociais e psicológicos

O impacto do lipedema na saúde mental e na qualidade de vida não pode ser subestimado. O distúrbio físico crônico frequentemente leva a desafios psicológicos, incluindo depressão, ansiedade e baixa autoestima, que são exacerbados pela dor crônica e pelas limitações físicas^4^ . Isso pode resultar em um ciclo vicioso de declínio da saúde mental e da qualidade de vida, enfatizando a necessidade de intervenções psicológicas e suporte social como parte integrante do tratamento do lipedema. A maioria das afirmações que seguiram essa proposição foi prontamente aceita na Fase 2 do Consenso.

A hipótese de uma correlação entre lipedema e transtorno de déficit de atenção e hiperatividade (TDAH) é intrigante e sugere uma complexidade neuropsiquiátrica potencialmente subjacente ao lipedema^61^ . A impulsividade, um sintoma comum em indivíduos com TDAH, pode também se manifestar em pacientes com lipedema, impactando a gestão da doença e a tomada de decisões sobre tratamentos, incluindo a cirurgia. É imperativo que a impulsividade seja adequadamente gerenciada antes de procedimentos cirúrgicos, para garantir que as decisões dos pacientes estejam alinhadas com os resultados desejados e que eles estejam preparados para o pós-operatório. A associação do lipedema com outros transtornos psiquiátricos reforça a necessidade de avaliação e suporte psicológico integrados ao plano de tratamento^42,61-64^ .

O apoio psicológico e social é fundamental para abordar os desafios psicossociais impostos pelo lipedema. Questões de imagem corporal e o estigma associado ao aumento de peso podem ser devastadores. Estratégias de enfrentamento, incluindo terapia cognitivo-comportamental e grupos de apoio, podem ser vitais para ajudar os pacientes a lidar com o fardo emocional e sintomático do lipedema, promovendo o bem-estar mental e facilitando a adaptação à doença^42,61-64^ .

A dificuldade em encontrar roupas que acomodem o aumento das extremidades é uma preocupação prática e significativa para pacientes com lipedema, muitas vezes levando ao aumento do estresse e a um impacto na autoimagem. Isso destaca a necessidade de considerações ergonômicas e de *design* na moda e vestuário, assim como de orientação sobre adaptações e recursos personalizados que possam auxiliar no conforto e na aceitação do próprio corpo^70-73^ .

A autoconsciência e a educação sobre o lipedema são componentes críticos para o autocuidado eficaz. O conhecimento sobre a doença permite que os pacientes reconheçam os sintomas precocemente, busquem intervenções apropriadas e adotem práticas de manejo em casa, o que pode prevenir a progressão da doença e melhorar a qualidade de vida. Além disso, a educação pode capacitar os pacientes a se tornarem defensores de suas próprias necessidades de saúde, promovendo a busca por apoio e recursos adequados.

### Classificação

Existem diferentes formas de classificação do lipedema: por características anatômicas ou por estágio de progressão. As afirmações sobre essas classificações receberam alta concordância na Fase 3 deste Consenso. A classificação do lipedema em estágios fornece um quadro útil para entender a progressão da doença, mas pode não capturar completamente a variabilidade individual dos sintomas. Por exemplo, dois pacientes no mesmo estágio da doença podem relatar níveis de dor e incapacidade muito diferentes^21^ . Isso ressalta a importância de uma avaliação holística do paciente, que considere não apenas a aparência física, mas também a presença de comorbidades, o estado inflamatório e o impacto psicossocial da doença^10,12,92^ .

As classificações baseadas na localização do lipedema são ferramentas importantes para os profissionais de saúde, pois ajudam a mapear a extensão da doença e a planejar o tratamento. No entanto, essa abordagem pode, às vezes, simplificar demais a complexidade dos casos individuais, não levando em conta a totalidade das manifestações do lipedema, como dor e qualidade de vida. Assim, enquanto as classificações clínicas fornecem uma estrutura para a identificação e o manejo do lipedema, elas devem ser usadas em conjunto com uma avaliação clínica detalhada^10,12,49,78,92^ .

A estratificação do lipedema em estágios tem implicações diretas para o tratamento, que pode variar desde o manejo conservador até intervenções cirúrgicas. A cirurgia, como a lipoaspiração, é frequentemente indicada nos estágios avançados da doença, quando há uma restrição significativa da mobilidade e qualidade de vida. No entanto, a decisão de prosseguir com a cirurgia deve ser cuidadosamente considerada e baseada em uma avaliação individual abrangente, incluindo o desejo do paciente, expectativas realistas e a avaliação dos riscos e benefícios^10,12,77^ .

Em suma, embora a classificação em estágios seja uma parte valiosa do manejo do lipedema, a complexidade da doença requer uma abordagem personalizada que leve em consideração a experiência subjetiva do paciente e o impacto global da doença na sua vida.

### Etiologia, patogênese e fisiopatologia

O lipedema é reconhecido como uma doença crônica e sistêmica, o que significa que é se trata de uma condição de longa duração que afeta vários sistemas do corpo. Como uma doença crônica, o lipedema exige um manejo contínuo e pode ter um curso progressivo, frequentemente levando ao aumento do tecido adiposo e a sintomas associados, como dor, sensibilidade e inchaço^21,32^ . A natureza sistêmica do lipedema indica que, embora os sintomas sejam mais visíveis nos membros inferiores, os efeitos da doença podem ser mais abrangentes, possivelmente impactando os sistemas circulatório e linfático e contribuindo para uma inflamação crônica no corpo^40,82,98,109,110^ .

Além disso, há evidências que apontam para um componente hereditário no lipedema. Vários estudos sugerem uma predisposição genética, com a doença frequentemente aparecendo em famílias, sugerindo um padrão de herança. No entanto, a genética exata do lipedema ainda não é completamente compreendida e acredita-se que seja poligênica, ou seja, envolve a contribuição de vários genes. Além dos fatores genéticos, elementos ambientais e o estilo de vida também podem influenciar a manifestação e a severidade do lipedema^25,111^ .

Reconhecendo o lipedema como uma doença crônica e sistêmica com possíveis raízes hereditárias, é fundamental desenvolver estratégias de tratamento eficazes e fornecer o suporte adequado aos pacientes^9,10,25^ . Essa compreensão também reforça a necessidade de pesquisa contínua para entender melhor a patogênese da doença e desenvolver abordagens de tratamento personalizadas, baseadas na genética e nos mecanismos biológicos subjacentes.

A patogênese do lipedema é multifatorial, com potencial ligação a anormalidades do tecido conjuntivo, como evidenciado pela associação com a síndrome de Ehlers-Danlos e questões de hipermobilidade articular^26,37,88^ . O distúrbio é marcado por um acúmulo atípico de tecido adiposo nas extremidades, poupando o tronco e frequentemente afetando os membros superiores de maneira simétrica e bilateral^10^ .

A inflamação, as alterações hormonais e o aumento do fluido extracelular no tecido adiposo são fatores identificados como contribuintes para a patogênese do lipedema. Adicionalmente, a disfunção no sistema microvascular pode desempenhar um papel central no desenvolvimento da condição^84,112,113^ . Embora o lipedema não seja causado pela obesidade, e vice-versa, a obesidade pode exacerbar os sintomas do lipedema e até desencadear sua manifestação em indivíduos predispostos. A linfoestase, ou estagnação linfática, pode ocorrer em qualquer fase do lipedema, mas tende a ser mais prevalente nos estágios mais avançados da doença^19,47,82,83,109,110^ .

A compreensão completa dessas interações e o desenvolvimento de estratégias de tratamento eficazes são áreas que ainda requerem investigação significativa. É fundamental reconhecer que o lipedema e a obesidade, embora possam ocorrer concomitantemente, têm etiologias distintas e, como tal, necessitam de abordagens de diagnóstico e manejo diferenciadas.

### Abordagem multidisciplinar

O lipedema é reconhecido como uma doença crônica e progressiva do tecido adiposo, frequentemente subdiagnosticada e mal compreendida. Caracteriza-se por um acúmulo simétrico de gordura nos membros, geralmente poupando as mãos e os pés, e pode ser acompanhado de dor e edema. A patogênese do lipedema ainda não é completamente entendida, mas acredita-se que envolva fatores genéticos, hormonais e inflamatórios. Dada a sua natureza complexa e multifacetada, o tratamento do lipedema requer uma abordagem multiprofissional, que inclua não apenas a gestão dos sintomas físicos, mas também o suporte psicológico, enfatizando a necessidade de uma equipe diversificada de profissionais de saúde. Diante dessa situação, as afirmações sobre a abordagem multidisciplinar foram muito bem aceitas já na Fase 2 do Consenso.

A abordagem multidisciplinar é fundamental no tratamento do lipedema, uma vez que diferentes especialistas podem contribuir com suas competências específicas para um plano de tratamento holístico. Cirurgiões vasculares podem gerenciar complicações linfáticas associadas, enquanto endocrinologistas ajudam a otimizar o perfil hormonal e metabólico dos pacientes. Ortopedistas podem ser consultados para lidar com as questões musculoesqueléticas decorrentes do excesso de peso nos membros, enquanto cirurgiões plásticos podem realizar procedimentos para reduzir o acúmulo de gordura e melhorar a funcionalidade e a estética. Além disso, psiquiatras e profissionais de saúde mental são essenciais para o acompanhamento dos aspectos emocionais e psicológicos, frequentemente negligenciados. Ginecologistas também desempenham um papel importante, particularmente no manejo de mulheres com lipedema que apresentam problemas hormonais e questões ginecológicas concomitantes^48-50^ .

A conscientização sobre o lipedema é crucial para melhorar o reconhecimento e tratamento da doença. Muitos pacientes passam anos sem um diagnóstico ou recebem diagnósticos incorretos, como obesidade ou linfedema. Isso pode levar a tratamentos ineficazes e a um aumento do estigma e da frustração dos pacientes. Portanto, a educação continuada dos profissionais de saúde e a sensibilização da sociedade são passos essenciais para mitigar esses problemas e promover uma abordagem terapêutica mais eficaz e compassiva^10,61,78^ .

O exercício físico é um componente importante no manejo do lipedema, ajudando na melhoria da mobilidade, na redução do risco de complicações, como o linfedema, e na manutenção da saúde cardiovascular. No entanto, a atividade física deve ser adaptada para cada indivíduo, considerando a dor e a mobilidade. Um profissional de educação física com conhecimento específico sobre o lipedema pode elaborar programas de exercícios que minimizem o risco de lesões e maximizem os benefícios terapêuticos, considerando as limitações e necessidades específicas de cada paciente^54,55^ .

Um aspecto mais controverso, mas ainda com alta concordância e evidência, diz respeito à relação entre carboidratos e proteínas na alimentação, que foi incluído na Fase 3 do Consenso. É fundamental que nutricionistas, nutrólogos, endocrinologistas e todos os profissionais envolvidos no tratamento do lipedema tenham acesso a programas de nutrição específicos e realizem uma abordagem consensual entre a equipe e o paciente, uma vez que o desacordo entre os profissionais dificulta a já difícil adesão a programas alimentares^86^ .

### Tratamento

O tratamento do lipedema envolve abordagens em múltiplos campos, abrangendo desde nutrição e exercícios até cuidados clínicos e intervenções cirúrgicas. Ainda há muitas controvérsias nesse tema, mas há um consenso de que o tratamento não invasivo ou conservador deve ser o primeiro passo e uma parte essencial do controle da doença. A cirurgia não deve preceder o tratamento clínico conservador, que continua a ser fundamental mesmo após eventual procedimento cirúrgico.

### Tratamento conservador

O tratamento conservador do lipedema, uma doença crônica e sistêmica, visa fundamentalmente à melhoria da qualidade de vida do paciente através do alívio dos sintomas e da desaceleração da progressão da doença. O manejo adequado da condição deve ser holístico e multidisciplinar, integrando mudanças no estilo de vida, como uma nutrição balanceada e atividade física regular de baixo impacto, para ajudar a aliviar a dor e a sensibilidade que caracterizam o lipedema. Terapias de compressão e abordagens fisioterapêuticas, como a terapia física descongestionante complexa (CTD), têm se mostrado eficazes na redução do desconforto e do edema, embora a variação individual na resposta ao tratamento seja comum^30,82,101^ .

Além disso, o controle do peso corporal é essencial, especialmente quando o lipedema está associado à obesidade, uma condição que pode agravar os sintomas inflamatórios. As estratégias para alcançar um peso saudável devem focar em medidas antropométricas, como a proporção cintura-altura, para avaliar os riscos associados ao excesso de gordura corporal. A progressão dos sintomas do lipedema pode ser atenuada pela adoção de um estilo de vida saudável e um tratamento adequado, que inclui evitar episódios inflamatórios que podem precipitar a progressão da doença^59,60^ .

Para monitorar a eficácia das intervenções terapêuticas, recomenda-se o uso de instrumentos de avaliação da qualidade de vida, como o Questionário de Avaliação de Sintomas de Lipedema (QuASiL) ou o Short Form 36 Health Survey (SF-36). Esses instrumentos permitem uma avaliação contínua e objetiva do impacto das terapias no bem-estar do paciente, possibilitando ajustes no plano de tratamento conforme necessário para otimizar os resultados^92^ .

Por fim, é crucial que as terapias propostas sejam baseadas em evidências científicas sólidas. Não se deve incentivar os pacientes a se submeterem a múltiplos tratamentos custosos que não apresentam comprovação clara de eficácia. A integridade do tratamento do lipedema depende do uso consciente e fundamentado de recursos terapêuticos, sempre com o objetivo de proporcionar o melhor cuidado possível aos pacientes.

### Terapia compressiva e exercícios físicos

Dentro do espectro terapêutico do lipedema, a massagem manual se destaca como uma técnica valiosa, capaz de promover o alívio sintomático do inchaço e da dor. Essa abordagem terapêutica é fundamentada na melhoria da circulação e na estimulação do fluxo linfático, proporcionando benefícios tanto físicos quanto psicológicos aos pacientes^30,82,101^ .

A CDT é um pilar fundamental no tratamento do lipedema, mesmo nas fases iniciais da doença. Composta por várias estratégias, como cuidados com a pele, massagem linfática manual, exercícios e compressão, a CDT pode proporcionar um controle significativo sobre a progressão do lipedema, ajudando a prevenir a acumulação de líquidos e o avanço do inchaço^56-58^ .

Atividades físicas de baixo impacto, como exercícios aquáticos, caminhada e ioga, são recomendadas para a manutenção da mobilidade e o manejo do peso em pacientes com lipedema. Essas práticas oferecem o benefício adicional de serem gentis para as articulações, o que é especialmente importante em uma condição que pode causar dor e fragilidade articular. O fortalecimento muscular, particularmente dos músculos posteriores da coxa, demonstrou ser uma estratégia eficaz no manejo do lipedema, contribuindo para o alívio dos sintomas e para a melhoria da funcionalidade e qualidade de vida dos pacientes^54,55^ .

Com a progressão do lipedema, o uso de terapias de compressão, como meias elásticas, pode se tornar desafiador devido à desproporção dos membros e ao desconforto potencial. Por isso, a personalização desses dispositivos é crucial para assegurar a eficácia do tratamento e o conforto do paciente, sobretudo durante episódios de inflamação acentuada^30,82,101^ .

A prática do autocuidado é um componente essencial no manejo do lipedema. Técnicas como a automassagem com escovas de cerdas macias, o uso de plataformas vibratórias e a aplicação regular de meias elásticas podem oferecer autonomia ao paciente e um controle adicional sobre os sintomas, reforçando as medidas de tratamento aplicadas pelos profissionais de saúde^10,107,108^ .

### Orientação psicológica e nutricional

As práticas de gerenciamento do estresse, como *mindfulness* e técnicas de respiração, são reconhecidas por sua capacidade de melhorar o bem-estar geral. No contexto do lipedema, elas podem ter um papel valioso, não apenas aliviando o estresse psicológico, mas também mitigando a dor crônica e facilitando a adesão ao tratamento – aspectos frequentemente desafiadores para os pacientes^61,64^ .

A orientação nutricional é uma pedra angular no manejo do lipedema, tendo um impacto direto na capacidade dos pacientes de gerenciar o peso e otimizar a saúde geral. Dietas balanceadas podem ajudar a reduzir a inflamação sistêmica e os sintomas associados ao lipedema, além de potencialmente aumentar a eficácia de outras intervenções terapêuticas. Dietas específicas, como as anti-inflamatórias e cetogênicas, têm sido exploradas em relação ao lipedema e podem oferecer benefícios sintomáticos a alguns pacientes. Tais abordagens dietéticas devem ser consideradas cuidadosamente e adaptadas individualmente, dada a variabilidade de resposta entre os indivíduos^51,52,95,100,114-117^ .

A vitamina D, um nutriente com múltiplas funções biológicas, incluindo a modulação do sistema imunológico e da inflamação, é frequentemente encontrada em níveis deficientes em mulheres com lipedema. Portanto, a monitorização e a suplementação adequadas são essenciais para garantir a saúde óssea e possivelmente influenciar positivamente a progressão do lipedema. A adequação dos níveis de vitamina D pode desempenhar um papel no alívio dos sintomas e na prevenção de complicações associadas à condição^105,106^ .

### Intervenção cirúrgica

As intervenções cirúrgicas no contexto do lipedema devem ser consideradas como parte de um espectro abrangente de cuidados e executadas em centros especializados, com proficiência tanto em procedimentos conservadores quanto cirúrgicos. A experiência do centro médico é crucial para garantir uma abordagem integral e personalizada no tratamento do lipedema.

A indicação cirúrgica para o lipedema deve ser uma decisão colaborativa entre o médico clínico, o cirurgião e a paciente, com o entendimento claro de que a prioridade é a melhora funcional, em particular a mobilidade, seguida pela melhoria dos sintomas. A decisão cirúrgica não deve ser precipitada, considerando que não há, ainda, seguimento a longo prazo ou acompanhamento em número significativo de pacientes abrangendo todos os aspectos do lipedema. Resultados estéticos, embora possam ser um benefício secundário, não devem ser o foco principal^5,10,102^ .

Pacientes mais jovens e em estágios iniciais do lipedema frequentemente apresentam resultados positivos após a cirurgia; entretanto, essas são as mesmas que obtêm melhores resultados com o tratamento conservador. O tratamento conservador possui benefícios universais e deve ser considerado antes de procedimentos cirúrgicos, os quais devem ser cuidadosamente avaliados quanto aos seus efeitos funcionais e potenciais impactos na fertilidade, que atualmente ainda são incertos^5,19,53,77^ .

A cirurgia, como uma opção de tratamento para o lipedema, não deve ser realizada de forma precipitada e é geralmente aconselhável explorar totalmente as opções de tratamento clínico por pelo menos 1 ano antes de considerar o procedimento. A compreensão da paciente sobre sua condição e a resposta ao tratamento clínico são essenciais para orientar essa decisão. Em casos de lipedema avançado, em que a mobilidade é significativamente afetada, a cirurgia pode ser considerada mais precocemente^5,10,102^ .

### Prevenção e prognóstico

É muito difícil, no momento, ter algum prognóstico ou definir ações preventivas certeiras para o lipedema. No entanto, é claro que alguns pontos sobre o assunto são consensuais, como evidenciado pela aceitação imediata de todas as assertivas na Fase 2 deste trabalho.

Quando o lipedema está associado à obesidade e a doenças metabólicas, a gestão do peso por meio de cirurgia bariátrica pode ser considerada uma etapa inicial antes de tratamentos específicos para o lipedema, como a lipoaspiração. A redução do peso pode diminuir a pressão sobre o sistema linfático e reduzir os sintomas relacionados ao lipedema^10,21,65-68^ .

A identificação precoce e a intervenção no lipedema são cruciais para prevenir a progressão da doença e melhorar o prognóstico^21^ . Tratamentos iniciados cedo podem estabilizar o acúmulo de gordura e prevenir complicações, como linfoestase e fibrose.

A cirurgia bariátrica pode resultar em perda de peso significativa em pacientes com obesidade, mas é importante notar que os sintomas do lipedema, como o acúmulo característico de gordura e a dor, podem não ser resolvidos apenas com a perda de peso e podem exigir abordagens de tratamento adicionais^25,40,41^ .

O diagnóstico e tratamento tardios do lipedema podem levar a uma maior carga sintomática e impactar adversamente a saúde mental e a qualidade de vida dos pacientes. Essa situação pode ocorrer devido à progressão da doença para estágios mais avançados, que são mais difíceis de tratar e podem resultar em maior incapacidade e dor.

### Futuro, pesquisa e desenvolvimento

Embora o lipedema tenha sido descrito clinicamente pela primeira vez em 1940, somente nos últimos anos houve um aumento significativo no volume de publicações sobre o tema. A necessidade de mais pesquisas para esclarecer os mecanismos biológicos do lipedema é fundamental. O entendimento aprofundado da etiologia da doença é crucial para o desenvolvimento de critérios de diagnóstico precisos e para a criação de terapias mais eficazes e personalizadas. Isso é particularmente importante quando consideramos a complexidade dos sinais e sintomas do lipedema e a variedade de respostas ao tratamento entre os pacientes.

Os estudos de longo prazo são indispensáveis não apenas para verificar a segurança e a eficácia das intervenções terapêuticas, mas também para entender os efeitos a longo prazo desses tratamentos, especialmente os cirúrgicos, que podem ter impactos significativos tanto físicos quanto metabólicos e psicológicos. O seguimento a longo prazo ajudará a esclarecer o curso da doença e a eficácia sustentada das diferentes modalidades de tratamento.

A colaboração entre pacientes, pesquisadores, médicos e grupos de apoio é essencial para o avanço do conhecimento sobre o lipedema. Essas parcerias podem melhorar o *design* e a implementação de estudos, garantir a inclusão das perspectivas dos pacientes e acelerar a aplicação de descobertas clínicas^69^ .

A possível ligação entre o lipedema e condições endócrinas, como o hipotireoidismo, indica que podem existir mecanismos sistêmicos subjacentes comuns entre essas condições que necessitam de mais investigação. Isso pode levar a uma melhor compreensão de como o lipedema afeta e é afetado por outras condições sistêmicas^12,55,69^ .

Por fim, a associação entre o lipedema e a presença de varizes destaca a importância de considerar a interação entre várias condições vasculares. Isso pode ter implicações para o tratamento, indicando a necessidade de uma abordagem integrada para o manejo dessas condições.

Este Consenso representa um avanço significativo na compreensão do lipedema no Brasil, marcando o início de uma jornada em busca de maior clareza e melhores práticas no diagnóstico e tratamento. Reconhecemos que há um longo e desafiador caminho pela frente, mas esse esforço conjunto fornece uma base sólida para futuras investigações e melhorias no cuidado aos pacientes com lipedema.

## CONCLUSÃO

O primeiro Consenso Brasileiro de Lipedema destaca a complexidade dessa condição e a necessidade de uma abordagem multidisciplinar para o tratamento. Embora tenhamos alcançado um entendimento significativo, ainda carecemos de um consenso absoluto e de um protocolo de tratamento definido. O tratamento conservador deve ser priorizado, com a cirurgia sendo considerada apenas após esgotadas essas opções. Este Consenso é um passo fundamental na melhoria do diagnóstico e tratamento do lipedema, mas reconhecemos a necessidade contínua de pesquisa e desenvolvimento de estratégias mais eficazes para o manejo da doença.
